# Thin endometrium transcriptome analysis reveals a potential mechanism of implantation failure

**DOI:** 10.1002/rmb2.12030

**Published:** 2017-04-09

**Authors:** Ryo Maekawa, Toshiaki Taketani, Yumiko Mihara, Shun Sato, Maki Okada, Isao Tamura, Kosuke Jozaki, Takuya Kajimura, Hiromi Asada, Hiroshi Tamura, Akihisa Takasaki, Norihiro Sugino

**Affiliations:** ^1^ Department of Obstetrics and Gynecology Yamaguchi University Graduate School of Medicine Ube Japan; ^2^ Department of Obstetrics and Gynecology Saiseikai Shimonoseki General Hospital Shimonoseki Japan

**Keywords:** implantation failure, infertility, oxidative stress, thin endometrium, uterine natural killer cells

## Abstract

**Aim:**

Although a thin endometrium has been well recognized as a critical factor in implantation failure, little information is available regarding the molecular mechanisms. The present study investigated these mechanisms by using genome‐wide mRNA expression analysis.

**Methods:**

Thin and normal endometrial tissue was obtained from a total of six women during the mid‐luteal phase of the menstrual cycle. The transcriptomes were analyzed with a microarray. Differentially expressed genes were classified according to Gene Ontology (GO) terms and Kyoto Encyclopedia of Genes and Genomes (KEGG) pathways.

**Results:**

The study identified 318 up‐regulated genes and 322 down‐regulated genes in the thin endometrium, compared to the control endometrium. The GO and KEGG pathway analyses indicated that the thin endometrium possessed aberrantly activated immunity and natural killer cell cytotoxicity that was accompanied by an increased number of inflammatory cytokines, such as *IFN‐*γ. Various genes that were related to metabolism and anti‐oxidative stress were down‐regulated in the thin endometrium.

**Conclusion:**

Implantation failure in the thin endometrium appears to be associated with an aberrantly activated inflammatory environment and aberrantly decreased response to oxidative stress.

## 1 Introduction

Adequate growth of the endometrium is indispensable for a successful pregnancy. Women with thin endometria have lower pregnancy rates, largely related to implantation failure.^1^, ^2^, ^3^, ^4^, ^5^, ^6^ The authors recently found high blood impedance in the uterine radial artery in patients with a thin endometrium^4^ and that vitamin E, L‐arginine, and sildenafil citrate treatments, which increase the blood flow of the uterine radial artery, helped to thicken the endometrium.^7^ This suggests that a low level of blood flow to the endometrium reduces its thickness, although it remains unclear why this would result in implantation failure. In order to answer this question, the authors compared the transcriptomes of thin and normal endometrial tissues with a microarray.

## 2 Materials and Methods

### 2.1 Tissue sampling

In total, six women with a history of infertility were recruited into the study. All the patients were diagnosed with unexplained infertility after excluding any obvious cause of infertility, such as uterine fibroid, endometriosis, tubal obstruction, and uterine malformation. The patients were classified into two groups, based on the endometrial thickness and level of blood flow impedance in the uterine radial artery. The endometrial thickness was measured at the maximal distance between each myometrial–endometrial interface by using vaginal ultrasonography in the mid‐luteal phase. The level of blood flow impedance in the uterine radial artery was measured as a resistance index with a pulsed Doppler. The cut‐off value of the endometrial thickness and level of blood flow impedance were defined as <8 mm and ≥0.81 mm, respectively, based on the authors' previous studies.^4^, ^7^, ^8^ Three patients had a normal‐thickness endometrium (endometrial thickness ≥8 mm) and three patients had a thin endometrium (Thin; endometrial thickness <8 mm) (Table 1). The endometrial thickness of the normal‐thickness endometrium group and the thin‐endometrium group was 9.53±0.65 and 6.33±0.68 mm, respectively. The difference in the endometrial thickness was significant (*P*=.0042). A high level of blood flow impedance in the uterine radial artery was confirmed only in the patients with a thin endometrium (0.86±0.04 vs 0.76±0.03; *P*=.027). The differences between the groups in age (31.7±3.21 vs 31.3±5.13 years), menstrual cycles (3.00±4.58 and 28.3±1.52 days), and serum levels of estradiol (171.3±16.6 vs 182.1±72.1 pg/mL) and progesterone (15.4±2.3 vs 21.5±7.6 μg/mL) were not significant. The endometrial tissue was obtained during the mid‐luteal phase of the menstrual cycle. Samples of endometrial curettings were washed with saline to remove the blood, immersed in liquid nitrogen, and stored at −80°C until RNA extraction.

**Table 1:** Cases of normal‐thickness and thin endometrium

Group	Age (years)	Menstrual cycle (days)	Endometrial thickness (mm)	Sampling date (from LMP)	Estradiol (pg/mL)	Progesterone (μg/mL)	Blood flow impedance (RI)
Control 1	34	26	9.5	20	166.0	18.0	0.823
Control 2	33	29	1.2	22	189.9	14.7	0.876
Control 3	28	35	8.9	29	158.0	13.6	0.893
Thin 1	37	27	7.1	20	247.5	26.4	0.759
Thin 2	30	30	5.8	22	193.9	25.3	0.750
Thin 3	27	28	6.1	21	104.8	12.7	0.781

Control, normal‐thickness endometrium; LMP, last menstrual period; thin, thin endometrium.

### 2.2 Transcriptome analysis

The total RNAs were isolated from the tissues by using TRIzol reagent (Invitrogen, Carlsbad, CA, USA) and they were reverse‐transcribed by using a QuantiTect Reverse Transcription Kit (Qiagen, Valencia, CA, USA), according to the manufacturer's protocol. The transcriptome analysis gene expression was analyzed by using a GeneChip Human Genome U133 Plus 2.0 Array (Affymetrix, Santa Clara, CA, USA) that contained 54 120 probes supporting 18 599 genes, as previously reported.^9^, ^10^ Briefly, the target cDNA was prepared from 200 ng of total RNA with the Ambion WT Expression kit (Ambion, Austin, TX, USA) and the Affymetrix GeneChip WT Terminal Labeling kit (Affymetrix). Hybridization to the microarrays, washing, staining, and scanning were performed by using the GeneChip system (Affymetrix), which was composed of the Scanner 3000 7G Workstation Fluidics 450 (Affymetrix) and the Hybridization Oven 645 (Affymetrix). The scanned image data were processed by using a gene expression analysis with the Partek Genomics Suite 6.5 software program (Partech, Munster, Germany). Then, 2000 randomly selected genes were used for the hierarchical clustering analysis and principal component analysis (PCA). Those genes whose expressions in the thin and normal endometrium differed by at least a factor of 2 and that had a false discovery rate of <.05 were judged as showing a significant difference.

### 2.3 Bioinformatics

A hierarchical clustering and a PCA were conducted in R v. 3.2.4.^11^ DAVID Bioinformatics Resources v. 6.7 (http://david.abcc.ncifcrf.gov/home.jsp) was used to determine whether the functional annotation of the differentially expressed genes was enriched for specific Gene Ontology (GO) terms and Kyoto Encyclopedia of Genes and Genomes (KEGG) pathways.^12^ Statistical significance was assessed with a modified Fisher's exact test. In the GO and KEGG analyses, *P*<.01 and *P*<.05, respectively, were considered to indicate significant enrichment. All the information from the GO and KEGG pathway analyses is shown in Tables S1‐S4.

## 3 Results

### 3.1 Comparison of the whole mRNA expression profiles of the normal and thin endometrial tissues

**Figure 1:**
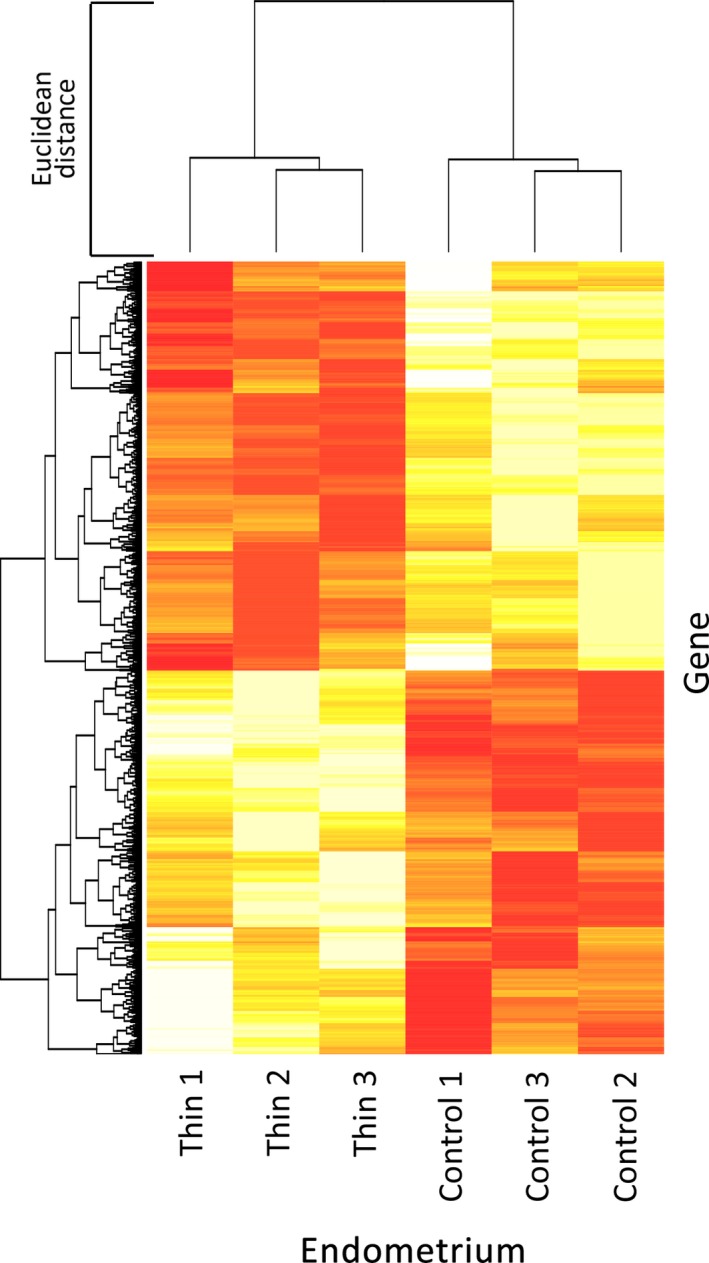
Heat map and hierarchical clustering of the mRNA expression profiles of patients with thin and normal (control) endometria. The mRNA expression profiles of the thin endometrium (Thin 1, Thin 2, and Thin 3) and the normal‐thickness endometrium (Control 1, Control 2, and Control 3) were compared. The *x*‐axis represents the samples and the *y*‐axis represents the gene clusters. The heat map in hierarchical clustering analysis indicates the mRNA expression levels from low (red) to high (yellow)

**Figure 2:**
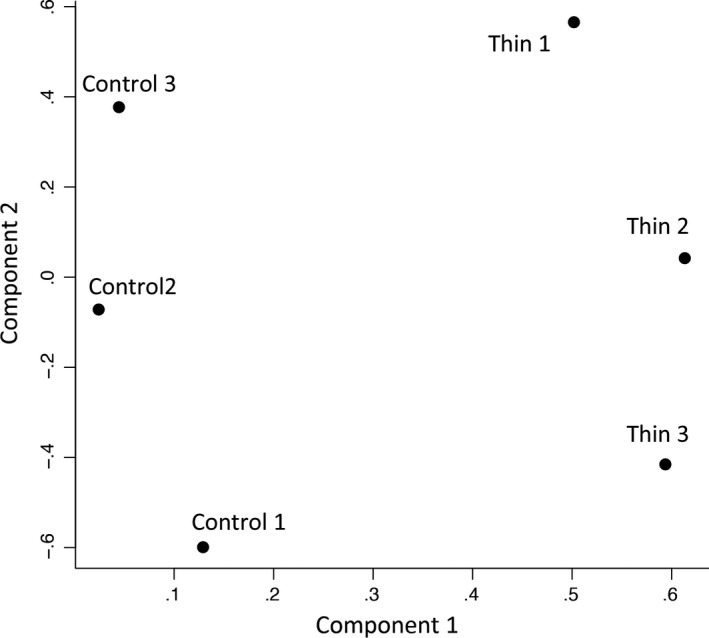
Principal component analysis of the mRNA expression profiles of patients with thin and normal (control) endometria. The mRNA expression profiles of the thin endometrium (Thin 1, Thin 2, and Thin 3) and the normal‐thickness endometrium (Control 1, Control 2, and Control 3) were compared. The *y*‐axis and the *x*‐axis show the principal component numbers, respectively

Figure 1 shows the mRNA expression profiles in the thin and normal endometrial tissues (n=3 for each). The hierarchical dendrogram clearly separated the thin and the control endometria. The PCA (Figure 2) also clearly separated the thin and the control endometria.

### 3.2 Gene Ontology and Kyoto Encyclopedia of Genes and Genomes pathway analyses of the up‐regulated genes in the thin endometrium

In the thin‐endometrium group, 318 genes were up‐regulated and 322 genes were down‐regulated, compared to the control endometrium group (Tables S5 and S6). The up‐regulated genes in the thin endometrium were related to immunity processes, such as the “response to external stimulus,” “defense response,” “leukocyte mediated immunity,” “immune response,” “immune effector process,” and “regulation of immune system process” (Tables 2 and S1). These GO terms included genes for interferon gamma (*IFN‐*γ), cytotoxic T‐lymphocyte proteinase 1 (*GZMA*) and 2 (*GZMB*), tumor necrosis factor ligand superfamily member 6 (*FASLG*), and tumor necrosis factor alpha (*TNF‐*α)‐induced genes, such as TNF‐α‐induced protein 2 (*TNFAIP2)* and 6 (*TNFAIP6*).

**Table 2:** Gene ontology analysis for the genes that were up‐regulated in the thin endometrium

Category	Term	Count	*P* value	Gene
GOTERM_BP_FAT	Response to external stimulus	70	1.16E‐09	*ARSB*,* FGF18*,* NRP1*,* MMP7*,***FASLG***,* PMAIP1*,* FOS*,* CD96*,* MYOCD*,* APOD*,* HPSE*,* RNASE7*,***IFNG***,* SLC2A1*,* CALCRL*,* CFI*,* LBP*,* MX1*,* ANGPT2*,* FCGR3B*,* F11*,* ZFP36*,* TRPM8*,* KIF5A*,* SOCS3*,* GNLY*,* PIM1*,* FOSB*,* PLAUR*,***TNFAIP6***,* SSTR2*,* THBD*,* ADM*,* IL20RB*,* TREM1*,* DPF3*,* PRF1*,* RBP1*,* ASS1*,* CCR1*,* STATH*,* CXCL2*,* GAST*,* TRDC*,* CCL4*,* IRAK3*,* RAC2*,* INPP5F*,* ARNTL2*,* BHLHE40*,* PTPRC*,* ST6GAL1*,* SLC8A1*,* VAV3*,* AIMP1*,* CFB*,* LMCD1*,* NR4A1*,* NR4A3*,* COTL1*,* PLAC8*,* SOD2*,* CORO1A*,* CD55*,* SLC7A2*,* CXCL13*,* CMTM7*,* CP*,* XCL1*,* HTR2A*
GOTERM_BP_FAT	Defense response	56	3.70E‐09	*KLRC2*,* IL19*,* PMAIP1*,* FOS*,* CD96*,* APOD*,* RNASE7*,***IFNG***,* VNN1*,* CALCRL*,* CFI*,* LBP*,* MX1*,* KLRD1*,* FCGR3B*,* DPP4*,* ZFP36*,* EGR1*,* NFKBIZ*,* SOCS3*,* GNLY*,***GZMB***,* CD84*,***TNFAIP6***,* OR2H2*,* CAMK4*,* ADM*,* IL20RB*,* TREM1*,* KIR3DL1*,* KIR2DL4*,* KIR3DL2*,* DPF3*,* PRF1*,* ASS1*,* CCR1*,* CXCL2*,* STATH*,* TRDC*,* C1S*,* CCL4*,* IRAK3*,* RASGRP1*,* BLNK*,* PTPRC*,* AIMP1*,* CFB*,* LMCD1*,* COTL1*,* PLAC8*,* CD55*,* CORO1A*,* SLC7A2*,* CXCL13*,* C1RL*,* XCL1*
GOTERM_BP_FAT	Leukocyte mediated immunity	21	1.62E‐07	*PTPRC*,* KLRC2*,***GZMB***,* TRDC*,* C1S*,* NR4A3*,* CD84*,* CD96*,* CORO1A*,* CD55*,* RAC2*,* CAMK4*,* IL20RB*,* RASGRP1*,***IFNG***,* C1RL*,* TREM1*,* CFI*,* XCL1*,* KLRD1*,* KIR3DL1*
GOTERM_BP_FAT	Immune response	50	1.99E‐07	*PRF1*,* KLRC2*,* ASS1*,* CCR1*,* IL19*,* CXCL2*,***FASLG***,* C1S*,* TRDC*,* ZEB1*,* CCL4*,* CD96*,* IRAK3*,* FOS*,* RAC2*,* RNASE7*,* RASGRP1*,***IFNG***,* VNN1*,* CFI*,* LBP*,* MX1*,* FCGR3B*,* KLRD1*,* BLNK*,* EGR1*,* PTPRC*,* ST6GAL1*,* VAV3*,* CFB*,* SOCS3*,***GZMA***,***GZMB***,* NR4A3*,* PRKCB*,* CTSW*,* CD84*,* CD38*,* CORO1A*,* CD55*,* MYO10*,* RGS1*,* IL20RB*,* ADM*,* CAMK4*,* CXCL13*,* C1RL*,* TREM1*,* XCL1*,* KIR3DL1*
GOTERM_BP_FAT	Immune effector process	34	2.07E‐07	*DPF3*,* PRF1*,* RBP4*,* KLRC2*,* PMAIP1*,* TRDC*,* C1S*,* CD96*,* IRAK3*,* RAC2*,* RASGRP1*,***IFNG***,* LBP*,* CFI*,* MX1*,* FCGR3B*,* KLRD1*,* PTPRC*,* VAV3*,* AIMP1*,* CFB*,* LMCD1*,***GZMB***,* NR4A3*,* CD84*,* MYO10*,* CD55*,* CORO1A*,* IL20RB*,* CAMK4*,* C1RL*,* TREM1*,* XCL1*,* KIR3DL1*
GOTERM_BP_FAT	Regulation of immune system process	46	1.96E‐06	*DPF3*,* RBP4*,* RBP1*,* CCR1*,* CXCL2*,* C1S*,* TRDC*,* ZEB1*,* CCL4*,* FOS*,* IRAK3*,* CD96*,* CDKN2A*,* RAC2*,* APOD*,* RASGRP1*,***IFNG***,* VNN1*,* LBP*,* CFI*,* FCGR3B*,* KLRD1*,* DPP4*,* ZFP36*,* PTPRC*,* VAV3*,* SOCS3*,* CFB*,* LMCD1*,* NR4A3*,* PRKCB*,* CD84*,* CD38*,* CD55*,* MYO10*,* CORO1A*,* CAMK4*,* IL20RB*,* SLC7A2*,* CXCL13*,* C1RL*,* TREM1*,* XCL1*,* KIR3DL1*,* KIR2DL4*,* KIR3DL2*
GOTERM_CC_FAT	Extracellular space	47	2.70E‐06	*RBP4*,* FGF18*,* NRP1*,* CXCL2*,* IL19*,* MMP7*,***FASLG***,* GAST*,* C1S*,* TRDC*,* SFN*,* CCL4*,* MTHFD2*,* APOD*,***IFNG***,* SLC2A1*,* LBP*,* CFI*,* ANGPT2*,* QSOX1*,* SRGN*,* F11*,* AIMP1*,* CFB*,* GNLY*,* HSPG2*,* LMCD1*,* IGFALS*,* COL25A1*,* CTSW*,* PROM1*,* MMP10*,***TNFAIP6***,* DKK1*,* THBD*,* ADM*,* SERPINB5*,* CXCL13*,* FABP3*,* C1RL*,* CMTM7*,* VCAN*,* IGFBP1*,* CP*,* XCL1*,***TNFAIP2***,* HABP2*
GOTERM_BP_FAT	Vasculature development	26	5.11E‐06	*FGFR2*,* ZFAND5*,* FGF18*,* NRP1*,* LEPR*,***FASLG***,* APOD*,* MYOCD*,* HPSE*,***IFNG***,* ROBO4*,* CALCRL*,* ANGPT2*,* THBS2*,* EGR1*,* VAV3*,* AIMP1*,* SOCS3*,* HSPG2*,* NR4A1*,* PRKCB*,* ADM*,* CXCL13*,* SIX1*,* HIF3A*,***TNFAIP2***
GOTERM_BP_FAT	Leukocyte activation	29	7.06E‐06	*PRF1*,* LEPR*,* ZEB1*,* TRDC*,* CDKN2A*,* RAC2*,* RASGRP1*,***IFNG***,* VNN1*,* LBP*,* DPP4*,* BLNK*,* EGR1*,* PTPRC*,* IL2RB*,* VAV3*,* IKZF1*,* DOCK8*,* NR4A3*,* PRKCB*,* CD84*,* CD38*,* CORO1A*,* CD55*,* IL20RB*,* CAMK4*,* SLC7A2*,* CMTM7*,* XCL1*
GOTERM_BP_FAT	Lymphocyte mediated immunity	16	1.17E‐05	*PTPRC*,* KLRC2*,***GZMB***,* TRDC*,* C1S*,* CD96*,* CORO1A*,* CD55*,* IL20RB*,* RASGRP1*,***IFNG***,* C1RL*,* CFI*,* XCL1*,* KLRD1*,* KIR3DL1*
GOTERM_BP_FAT	Inflammatory response	26	1.19E‐05	*ZFP36*,* NFKBIZ*,* AIMP1*,* ASS1*,* SOCS3*,* CFB*,* CCR1*,* IL19*,* CXCL2*,* CCL4*,* FOS*,* CD96*,***TNFAIP6***,* CD55*,* CAMK4*,* IL20RB*,* APOD*,* SLC7A2*,* CXCL13*,* RASGRP1*,* VNN1*,* LBP*,* CFI*,* CALCRL*,* XCL1*,* BLNK*
GOTERM_BP_FAT	Leukocyte migration	19	1.42E‐05	*VAV3*,* AIMP1*,* CCR1*,* CXCL2*,* DOCK8*,* CCL4*,* MMP1*,* SLC7A11*,* CD84*,* CORO1A*,* THBD*,* RAC2*,* APOD*,* CXCL13*,***IFNG***,* TREM1*,* LBP*,* XCL1*,* ANGPT2*
GOTERM_BP_FAT	Locomotion	45	1.59E‐05	*ARSB*,* ZFAND5*,* FGF18*,* NRP1*,* CCR1*,* CXCL2*,* FERMT1*,* CCL4*,* MMP1*,* DNAH6*,* NPHP4*,* RAC2*,* APOD*,***IFNG***,* ROBO4*,* INPP5F*,* LBP*,* DEPDC1B*,* ANGPT2*,* DPP4*,* PTPRC*,* SLC8A1*,* ST6GAL1*,* SATB2*,* S100P*,* VAV3*,* AIMP1*,* KIF5A*,* NR4A1*,* DOCK7*,* DOCK8*,* NR4A3*,* SLC7A11*,* PLAUR*,* CD84*,* MMP10*,***TNFAIP6***,* CORO1A*,* THBD*,* CXCL13*,* SIX1*,* CMTM7*,* VCAN*,* TREM1*,* XCL1*
GOTERM_BP_FAT	Cell activation	32	1.61E‐05	*PRF1*,* LEPR*,* ZEB1*,* TRDC*,* CDKN2A*,* RAC2*,* MYOCD*,* RASGRP1*,***IFNG***,* VNN1*,* LBP*,* DPP4*,* BLNK*,* EGR1*,* PTPRC*,* IL2RB*,* VAV3*,* IKZF1*,* DOCK8*,* NR4A3*,* SLC7A11*,* PRKCB*,* CD84*,* CD38*,* CORO1A*,* CD55*,* THBD*,* IL20RB*,* CAMK4*,* SLC7A2*,* CMTM7*,* XCL1*
GOTERM_BP_FAT	Regulation of response to external stimulus	30	1.68E‐05	*FGF18*,* DPF3*,* NRP1*,* CCR1*,* CXCL2*,* CCL4*,* RAC2*,* APOD*,* HPSE*,* INPP5F*,* CALCRL*,* CFI*,* LBP*,* FCGR3B*,* ANGPT2*,* ZFP36*,* F11*,* ST6GAL1*,* CFB*,* SOCS3*,* LMCD1*,* PLAUR*,***TNFAIP6***,* CD55*,* THBD*,* IL20RB*,* CXCL13*,* SLC7A2*,* TREM1*,* XCL1*
GOTERM_BP_FAT	Blood vessel development	24	1.89E‐05	*EGR1*,* FGFR2*,* FGF18*,* NRP1*,* VAV3*,* AIMP1*,* SOCS3*,* LEPR*,* HSPG2*,***FASLG***,* NR4A1*,* PRKCB*,* MYOCD*,* APOD*,* ADM*,* HPSE*,* SIX1*,***IFNG***,* ROBO4*,* HIF3A*,* CALCRL*,* THBS2*,***TNFAIP2***,* ANGPT2*
GOTERM_BP_FAT	Cardiovascular system development	32	3.29E‐05	*FGFR2*,* ZFAND5*,* FGF18*,* RBP4*,* NRP1*,* LEPR*,* PDLIM3*,***FASLG***,* ZIC3*,* APOD*,* MYOCD*,* HPSE*,***IFNG***,* ROBO4*,* CALCRL*,* ANGPT2*,* THBS2*,* EGR1*,* SLC8A1*,* VAV3*,* AIMP1*,* SOCS3*,* HSPG2*,* NR4A1*,* PRKCB*,* SOD2*,* DKK1*,* ADM*,* CXCL13*,* SIX1*,* HIF3A*,***TNFAIP2***
GOTERM_BP_FAT	Circulatory system development	32	3.29E‐05	*FGFR2*,* ZFAND5*,* FGF18*,* RBP4*,* NRP1*,* LEPR*,* PDLIM3*,***FASLG***,* ZIC3*,* APOD*,* MYOCD*,* HPSE*,***IFNG***,* ROBO4*,* CALCRL*,* ANGPT2*,* THBS2*,* EGR1*,* SLC8A1*,* VAV3*,* AIMP1*,* SOCS3*,* HSPG2*,* NR4A1*,* PRKCB*,* SOD2*,* DKK1*,* ADM*,* CXCL13*,* SIX1*,* HIF3A*,***TNFAIP2***
GOTERM_BP_FAT	Lymphocyte activation	25	3.54E‐05	*PRF1*,* LEPR*,* TRDC*,* ZEB1*,* CDKN2A*,* RAC2*,* RASGRP1*,***IFNG***,* VNN1*,* DPP4*,* BLNK*,* EGR1*,* PTPRC*,* IL2RB*,* VAV3*,* IKZF1*,* DOCK8*,* PRKCB*,* CD38*,* CD55*,* CORO1A*,* IL20RB*,* CAMK4*,* CMTM7*,* XCL1*
GOTERM_BP_FAT	Blood vessel morphogenesis	21	4.88E‐05	*FGFR2*,* FGF18*,* NRP1*,* VAV3*,* AIMP1*,* LEPR*,* HSPG2*,* NR4A1*,***FASLG***,* PRKCB*,* MYOCD*,* APOD*,* ADM*,* HPSE*,* SIX1*,* ROBO4*,* HIF3A*,* CALCRL*,* THBS2*,***TNFAIP2***,* ANGPT2*
GOTERM_BP_FAT	Angiogenesis	19	4.88E‐05	*FGFR2*,* FGF18*,* NRP1*,* VAV3*,* AIMP1*,* LEPR*,* HSPG2*,* NR4A1*,***FASLG***,* PRKCB*,* APOD*,* ADM*,* HPSE*,* ROBO4*,* HIF3A*,* CALCRL*,* THBS2*,***TNFAIP2***,* ANGPT2*
GOTERM_BP_FAT	Negative regulation of multicellular organismal process	33	6.30E‐05	*RBP4*,* NRP1*,* CCR1*,* STATH*,***FASLG***,* CD96*,* IRAK3*,* CDKN2A*,* APOD*,* MYOCD*,***IFNG***,* INPP5F*,* LBP*,* CALCRL*,* ANGPT2*,* THBS2*,* SRGN*,* NR2F1*,* ZFP36*,* F11*,* ANXA4*,* PLAUR*,* PLAC8*,* CD84*,* CD38*,* DKK1*,* THBD*,* ADM*,* IL20RB*,* CXCL13*,* SIX1*,* ID4*,* XCL1*
GOTERM_BP_FAT	Regulation of cell activation	21	7.32E‐05	*PTPRC*,* VAV3*,* ZEB1*,* TRDC*,* NR4A3*,* CD84*,* CD38*,* CORO1A*,* CD55*,* THBD*,* CDKN2A*,* RAC2*,* CAMK4*,* IL20RB*,* SLC7A2*,* RASGRP1*,***IFNG***,* VNN1*,* LBP*,* XCL1*,* DPP4*
GOTERM_BP_FAT	Cell motility	39	7.52E‐05	*ARSB*,* ZFAND5*,* FGF18*,* NRP1*,* CCR1*,* CXCL2*,* FERMT1*,* CCL4*,* MMP1*,* DNAH6*,* NPHP4*,* RAC2*,* APOD*,***IFNG***,* ROBO4*,* LBP*,* DEPDC1B*,* ANGPT2*,* DPP4*,* PTPRC*,* SLC8A1*,* SATB2*,* S100P*,* VAV3*,* AIMP1*,* NR4A1*,* DOCK7*,* DOCK8*,* SLC7A11*,* CD84*,* MMP10*,***TNFAIP6***,* CORO1A*,* THBD*,* CXCL13*,* SIX1*,* VCAN*,* TREM1*,* XCL1*
GOTERM_BP_FAT	Localization of cell	39	7.52E‐05	*ARSB*,* ZFAND5*,* FGF18*,* NRP1*,* CCR1*,* CXCL2*,* FERMT1*,* CCL4*,* MMP1*,* DNAH6*,* NPHP4*,* RAC2*,* APOD*,***IFNG***,* ROBO4*,* LBP*,* DEPDC1B*,* ANGPT2*,* DPP4*,* PTPRC*,* SLC8A1*,* SATB2*,* S100P*,* VAV3*,* AIMP1*,* NR4A1*,* DOCK7*,* DOCK8*,* SLC7A11*,* CD84*,* MMP10*,***TNFAIP6***,* CORO1A*,* THBD*,* CXCL13*,* SIX1*,* VCAN*,* TREM1*,* XCL1*
GOTERM_BP_FAT	Regulation of leukocyte activation	20	8.61E‐05	*PTPRC*,* VAV3*,* ZEB1*,* TRDC*,* NR4A3*,* CD84*,* CD38*,* CORO1A*,* CD55*,* CDKN2A*,* RAC2*,* CAMK4*,* IL20RB*,* SLC7A2*,* RASGRP1*,***IFNG***,* VNN1*,* LBP*,* XCL1*,* DPP4*
GOTERM_BP_FAT	Regulation of immune effector process	19	8.63E‐05	*PTPRC*,* RBP4*,* DPF3*,* CFB*,* LMCD1*,* NR4A3*,* CD84*,* CD96*,* IRAK3*,* CD55*,* RAC2*,* IL20RB*,* RASGRP1*,***IFNG***,* TREM1*,* CFI*,* LBP*,* XCL1*,* FCGR3B*
GOTERM_CC_FAT	Extracellular region part	88	9.24E‐05	*ARSB*,* FGF18*,* NRP1*,* THRB*,* FAM20A*,* IL19*,* MMP7*,***FASLG***,* AURKB*,* MMP1*,* MTHFD2*,* APOD*,* HPSE*,* RNASE7*,* DMKN*,* SLC2A1*,***IFNG***,* ROBO4*,* VNN1*,* CFI*,* LBP*,* DPP6*,* ANGPT2*,* FCGR3B*,* DPP4*,* F11*,* GSTT2B*,* GNLY*,* IGFALS*,* COL25A1*,* PADI1*,* CTSW*,* PLAUR*,* PRKCB*,* CD84*,* MMP10*,***TNFAIP6***,* CD38*,* THBD*,* CAMK4*,* ADM*,* SERPINB5*,* CYBRD1*,* VCAN*,* SLC38A1*,* COL24A1*,***TNFAIP2***,* PPFIA2*,* RBP4*,* ASS1*,* CXCL2*,* GAST*,* C1S*,* SFN*,* TRDC*,* CCL4*,* LAMB3*,* RAC2*,* THBS2*,* QSOX1*,* SRGN*,* PTPRC*,* GPR155*,* ST6GAL1*,* VAV3*,* S100P*,* AIMP1*,* CFB*,* LMCD1*,* HSPG2*,* COTL1*,* ANXA4*,* SOD2*,* PROM1*,* CORO1A*,* CD55*,* DKK1*,* C1ORF116*,* CXCL13*,* FABP3*,* C1RL*,* CMTM7*,* H3F3B*,* IGFBP1*,* CP*,* XCL1*,* FABP5*,* HABP2*
GOTERM_BP_FAT	Response to biotic stimulus	32	9.65E‐05	*PRF1*,* DPF3*,* ASS1*,* CXCL2*,* STATH*,***FASLG***,* PMAIP1*,* TRDC*,* CCL4*,* FOS*,* CD96*,* IRAK3*,* RNASE7*,***IFNG***,* LBP*,* MX1*,* FCGR3B*,* ZFP36*,* PTPRC*,* AIMP1*,* SOCS3*,* GNLY*,* LMCD1*,* COTL1*,* PLAC8*,* SOD2*,* CD55*,* THBD*,* ADM*,* CXCL13*,* TREM1*,* XCL1*
GOTERM_BP_FAT	Anatomical structure formation involved in morphogenesis	35	9.69E‐05	*FGFR2*,* RBP4*,* FGF18*,* NRP1*,* FAM20A*,* ABLIM3*,* LEPR*,* BBS9*,***FASLG***,* MTHFD1L*,* WDR74*,* LAMB3*,* APOD*,* HPSE*,* ROBO4*,* CALCRL*,* LMOD3*,* ANGPT2*,* THBS2*,* VAV3*,* AIMP1*,* HSPG2*,* NR4A1*,* NR4A3*,* PRKCB*,* PROM1*,* DUSP5*,* DKK1*,* ADM*,* CXCL13*,* ETS2*,* SIX1*,* HIF3A*,***TNFAIP2***,* DUSP6*
GOTERM_BP_FAT	Cell chemotaxis	14	1.00E‐04	*FGF18*,* NRP1*,* VAV3*,* CCR1*,* CXCL2*,* NR4A1*,* CCL4*,* CORO1A*,* RAC2*,* CXCL13*,***IFNG***,* LBP*,* TREM1*,* XCL1*
GOTERM_BP_FAT	Response to other organism	31	1.05E‐04	*PRF1*,* DPF3*,* ASS1*,* CXCL2*,* STATH*,***FASLG***,* PMAIP1*,* TRDC*,* CCL4*,* FOS*,* CD96*,* IRAK3*,* RNASE7*,***IFNG***,* LBP*,* MX1*,* FCGR3B*,* ZFP36*,* PTPRC*,* AIMP1*,* SOCS3*,* GNLY*,* LMCD1*,* COTL1*,* PLAC8*,* SOD2*,* THBD*,* ADM*,* CXCL13*,* TREM1*,* XCL1*
GOTERM_BP_FAT	Response to external biotic stimulus	31	1.05E‐04	*PRF1*,* DPF3*,* ASS1*,* CXCL2*,* STATH*,***FASLG***,* PMAIP1*,* TRDC*,* CCL4*,* FOS*,* CD96*,* IRAK3*,* RNASE7*,***IFNG***,* LBP*,* MX1*,* FCGR3B*,* ZFP36*,* PTPRC*,* AIMP1*,* SOCS3*,* GNLY*,* LMCD1*,* COTL1*,* PLAC8*,* SOD2*,* THBD*,* ADM*,* CXCL13*,* TREM1*,* XCL1*
GOTERM_BP_FAT	Cell proliferation	49	1.29E‐04	*FGFR2*,* RBP4*,* FGF18*,* NRP1*,* FERMT1*,***FASLG***,* PMAIP1*,* ZEB1*,* AURKB*,* SFN*,* FAM83B*,* CDKN2A*,* APOD*,* RAC2*,* MYOCD*,* HPSE*,***IFNG***,* MTCP1*,* CALCRL*,* QSOX1*,* DPP4*,* EGR1*,* ZFP36*,* PTPRC*,* ST6GAL1*,* VAV3*,* TRNP1*,* AIMP1*,* PIM1*,* NR4A1*,* DOCK7*,* NR4A3*,* DOCK8*,* PLAC8*,* SOD2*,* CD38*,* SSTR2*,* CORO1A*,* CD55*,* IL20RB*,* ADM*,* SERPINB5*,* SIX1*,* FABP3*,* H3F3B*,* ID4*,* XCL1*,* EMP1*,* HTR2A*
GOTERM_CC_FAT	Extracellular region	100	1.29E‐04	*ARSB*,* FGF18*,* NRP1*,* THRB*,* FAM20A*,* LEPR*,* IL19*,* MMP7*,***FASLG***,* AURKB*,* MMP1*,* MTHFD2*,* APOD*,* HPSE*,* DMKN*,* RNASE7*,* SLC2A1*,***IFNG***,* ROBO4*,* VNN1*,* LBP*,* CFI*,* DPP6*,* GFOD1*,* FCGR3B*,* ANGPT2*,* CSF2RA*,* DPP4*,* F11*,* GSTT2B*,***GZMA***,* GNLY*,* IGFALS*,* COL25A1*,* PADI1*,* PRKCB*,* PLAUR*,* CTSW*,* CD84*,* MMP10*,* CD38*,***TNFAIP6***,* PSG9*,* THBD*,* ADM*,* CAMK4*,* SERPINB5*,* CYBRD1*,* VCAN*,* SLC38A1*,* TREM1*,* COL24A1*,***TNFAIP2***,* FGFR2*,* PPFIA2*,* PRF1*,* RBP4*,* ASS1*,* STATH*,* CXCL2*,* GAST*,* C1S*,* SFN*,* TRDC*,* CCL4*,* FAM19A5*,* LAMB3*,* RAC2*,* GLIPR1*,* QSOX1*,* THBS2*,* SRGN*,* PTPRC*,* GPR155*,* ST6GAL1*,* S100P*,* VAV3*,* AIMP1*,* CFB*,* HSPG2*,* LMCD1*,* GRIA3*,* COTL1*,* ANXA4*,* SOD2*,* PROM1*,* CD55*,* CORO1A*,* C1ORF116*,* DKK1*,* CXCL13*,* FABP3*,* C1RL*,* CMTM7*,* H3F3B*,* CP*,* IGFBP1*,* XCL1*,* FABP5*,* HABP2*
GOTERM_BP_FAT	Regulation of cell proliferation	43	1.36E‐04	*FGFR2*,* RBP4*,* FGF18*,* NRP1*,* CXCL2*,***FASLG***,* SFN*,* PMAIP1*,* ZEB1*,* CDKN2A*,* RAC2*,* MYOCD*,* APOD*,* HPSE*,***IFNG***,* CHST11*,* CALCRL*,* QSOX1*,* DPP4*,* ZFP36*,* EGR1*,* PTPRC*,* ST6GAL1*,* VAV3*,* AIMP1*,* TRNP1*,* NR4A1*,* NR4A3*,* PLAC8*,* SOD2*,* CD38*,* SSTR2*,* CD55*,* CORO1A*,* IL20RB*,* ADM*,* SERPINB5*,* CXCL13*,* SIX1*,* FABP3*,* ID4*,* XCL1*,* HTR2A*
GOTERM_BP_FAT	Movement of cell or subcellular component	47	1.38E‐04	*ARSB*,* ZFAND5*,* DNAH10*,* FGF18*,* NRP1*,* CCR1*,* CXCL2*,* FERMT1*,* CCL4*,* MMP1*,* DNAH6*,* NPHP4*,* RAC2*,* APOD*,* RASGRP1*,***IFNG***,* ROBO4*,* INPP5F*,* VNN1*,* LBP*,* DEPDC1B*,* ANGPT2*,* DPP4*,* PTPRC*,* SLC8A1*,* SATB2*,* S100P*,* VAV3*,* AIMP1*,* KIF5A*,* NR4A1*,* DOCK7*,* DOCK8*,* NR4A3*,* SLC7A11*,* PLAUR*,* CD84*,* MMP10*,***TNFAIP6***,* CORO1A*,* THBD*,* SERPINB5*,* CXCL13*,* SIX1*,* VCAN*,* TREM1*,* XCL1*
GOTERM_MF_FAT	Serine‐type peptidase activity	14	1.52E‐04	*F11*,***GZMA***,* CFB*,* MMP7*,***GZMB***,* C1S*,* TMPRSS3*,* MMP1*,* MMP10*,* C1RL*,* CFI*,* DPP6*,* DPP4*,* HABP2*
GOTERM_BP_FAT	Regulation of immune response	29	1.58E‐04	*TRDC*,* C1S*,* FOS*,* CD96*,* IRAK3*,* RAC2*,* RASGRP1*,***IFNG***,* LBP*,* CFI*,* KLRD1*,* PTPRC*,* VAV3*,* SOCS3*,* CFB*,* NR4A3*,* PRKCB*,* CD84*,* CD38*,* CD55*,* MYO10*,* IL20RB*,* CXCL13*,* C1RL*,* TREM1*,* XCL1*,* KIR3DL1*,* KIR3DL2*,* KIR2DL4*
GOTERM_BP_FAT	Cell migration	35	1.68E‐04	*ARSB*,* ZFAND5*,* FGF18*,* NRP1*,* CCR1*,* CXCL2*,* FERMT1*,* CCL4*,* MMP1*,* RAC2*,* APOD*,***IFNG***,* LBP*,* DEPDC1B*,* ANGPT2*,* DPP4*,* PTPRC*,* SLC8A1*,* SATB2*,* S100P*,* VAV3*,* AIMP1*,* NR4A1*,* DOCK7*,* DOCK8*,* SLC7A11*,* CD84*,***TNFAIP6***,* CORO1A*,* THBD*,* CXCL13*,* SIX1*,* VCAN*,* TREM1*,* XCL1*
GOTERM_MF_FAT	Serine hydrolase activity	14	1.70E‐04	*F11*,***GZMA***,* CFB*,* MMP7*,***GZMB***,* C1S*,* TMPRSS3*,* MMP1*,* MMP10*,* C1RL*,* CFI*,* DPP6*,* DPP4*,* HABP2*
GOTERM_BP_FAT	Cell adhesion	45	1.76E‐04	*PPFIA2*,* ASS1*,* SNX5*,* CCR1*,* LEPR*,* FERMT1*,* CLDN10*,* SFN*,* ZEB1*,* CCL4*,* CD96*,* LAMB3*,* NPHP4*,* CDKN2A*,* RAC2*,* APOD*,* HPSE*,* RASGRP1*,***IFNG***,* VNN1*,* THBS2*,* ANGPT2*,* DPP4*,* EGR1*,* PTPRC*,* ST6GAL1*,* S100P*,* VAV3*,* AIMP1*,* MAGI1*,* IGFALS*,* DOCK8*,* NR4A3*,* SLC7A11*,* CD84*,***TNFAIP6***,* MYO10*,* CD55*,* CORO1A*,* CAMK4*,* IL20RB*,* CXCL13*,* VCAN*,* XCL1*,* HABP2*
GOTERM_BP_FAT	Biological adhesion	45	1.91E‐04	*PPFIA2*,* ASS1*,* SNX5*,* CCR1*,* LEPR*,* FERMT1*,* CLDN10*,* SFN*,* ZEB1*,* CCL4*,* CD96*,* LAMB3*,* NPHP4*,* CDKN2A*,* RAC2*,* APOD*,* HPSE*,* RASGRP1*,***IFNG***,* VNN1*,* THBS2*,* ANGPT2*,* DPP4*,* EGR1*,* PTPRC*,* ST6GAL1*,* S100P*,* VAV3*,* AIMP1*,* MAGI1*,* IGFALS*,* DOCK8*,* NR4A3*,* SLC7A11*,* CD84*,***TNFAIP6***,* MYO10*,* CD55*,* CORO1A*,* CAMK4*,* IL20RB*,* CXCL13*,* VCAN*,* XCL1*,* HABP2*
GOTERM_BP_FAT	Negative regulation of response to stimulus	39	1.91E‐04	*NKD2*,* NRP1*,* IL19*,* TMEM161A*,***FASLG***,* IRAK3*,* CD96*,* NPHP4*,* APOD*,* CHST11*,* INPP5F*,* VNN1*,* CALCRL*,* ANGPT2*,* F11*,* ZFP36*,* EGR1*,* PTPRC*,* ST6GAL1*,* SOCS3*,* NR4A3*,* RGS14*,* PRKCB*,* PLAUR*,* SOD2*,* CD84*,* DUSP5*,***TNFAIP6***,* CD55*,* RGS1*,* DKK1*,* THBD*,* IL20RB*,* ADM*,* CXCL13*,* HELB*,* IGFBP1*,* XCL1*,* DUSP6*
GOTERM_BP_FAT	Negative regulation of response to external stimulus	14	1.97E‐04	*ZFP36*,* F11*,* ST6GAL1*,* NRP1*,* SOCS3*,* PLAUR*,***TNFAIP6***,* THBD*,* IL20RB*,* APOD*,* CXCL13*,* INPP5F*,* CALCRL*,* ANGPT2*
GOTERM_MF_FAT	Serine‐type endopeptidase activity	13	2.08E‐04	*F11*,* MMP10*,* CFB*,***GZMA***,* C1RL*,* MMP7*,***GZMB***,* CFI*,* C1S*,* DPP4*,* MMP1*,* TMPRSS3*,* HABP2*
GOTERM_BP_FAT	Chemotaxis	21	2.57E‐04	*FGF18*,* ST6GAL1*,* NRP1*,* VAV3*,* AIMP1*,* KIF5A*,* CCR1*,* CXCL2*,* NR4A1*,* NR4A3*,* CCL4*,* PLAUR*,* CORO1A*,* RAC2*,* CXCL13*,***IFNG***,* CMTM7*,* TREM1*,* LBP*,* XCL1*,* ANGPT2*
GOTERM_BP_FAT	Neutrophil chemotaxis	8	2.61E‐04	*VAV3*,* RAC2*,* CXCL2*,***IFNG***,* TREM1*,* LBP*,* XCL1*,* CCL4*
GOTERM_BP_FAT	Taxis	21	2.62E‐04	*FGF18*,* ST6GAL1*,* NRP1*,* VAV3*,* AIMP1*,* KIF5A*,* CCR1*,* CXCL2*,* NR4A1*,* NR4A3*,* CCL4*,* PLAUR*,* CORO1A*,* RAC2*,* CXCL13*,***IFNG***,* CMTM7*,* TREM1*,* LBP*,* XCL1*,* ANGPT2*
GOTERM_BP_FAT	Regulation of cell–cell adhesion	17	2.64E‐04	*PTPRC*,* ASS1*,* NR4A3*,* ZEB1*,* CORO1A*,* CD55*,* MYO10*,* CDKN2A*,* RAC2*,* CAMK4*,* IL20RB*,* CXCL13*,* RASGRP1*,***IFNG***,* VNN1*,* XCL1*,* DPP4*
GOTERM_BP_FAT	Regulation of apoptotic process	39	2.88E‐04	*FGFR2*,* NRP1*,* IL19*,* TMEM161A*,***FASLG***,* AURKB*,* SFN*,* PMAIP1*,* CDKN2A*,* MYOCD*,***IFNG***,* CHST11*,* ROBO4*,* VNN1*,* PHLDA1*,* ZFP36*,* EGR1*,* ST6GAL1*,* IL2RB*,* VAV3*,* SOCS3*,***GZMA***,* BCL2A1*,* PIM1*,* NR4A1*,***GZMB***,* DOCK8*,* NR4A3*,* ANXA4*,* PLAUR*,* PLAC8*,* SOD2*,* CD38*,* CORO1A*,* ADM*,* SIX1*,* LGALS14*,* TNFAIP8*,* DUSP6*
GOTERM_BP_FAT	Cell killing	9	2.90E‐04	*PTPRC*,* CORO1A*,* RASGRP1*,* GNLY*,***IFNG***,***GZMB***,* TREM1*,* XCL1*,* KIR3DL1*
GOTERM_BP_FAT	Regulation of leukocyte cell–cell adhesion	15	2.95E‐04	*PTPRC*,* ASS1*,* NR4A3*,* ZEB1*,* CORO1A*,* CD55*,* CDKN2A*,* RAC2*,* CAMK4*,* IL20RB*,* RASGRP1*,***IFNG***,* VNN1*,* XCL1*,* DPP4*
GOTERM_BP_FAT	Natural killer cell mediated immunity	7	3.01E‐04	*CD96*,* KLRC2*,* CORO1A*,* RASGRP1*,***GZMB***,* KLRD1*,* KIR3DL1*
GOTERM_BP_FAT	Regulation of programmed cell death	39	3.49E‐04	*FGFR2*,* NRP1*,* IL19*,* TMEM161A*,***FASLG***,* AURKB*,* SFN*,* PMAIP1*,* CDKN2A*,* MYOCD*,***IFNG***,* CHST11*,* ROBO4*,* VNN1*,* PHLDA1*,* ZFP36*,* EGR1*,* ST6GAL1*,* IL2RB*,* VAV3*,* SOCS3*,***GZMA***,* BCL2A1*,* PIM1*,* NR4A1*,***GZMB***,* DOCK8*,* NR4A3*,* ANXA4*,* PLAUR*,* PLAC8*,* SOD2*,* CD38*,* CORO1A*,* ADM*,* SIX1*,* LGALS14*,* TNFAIP8*,* DUSP6*
GOTERM_BP_FAT	Cell death	49	3.99E‐04	*FGFR2*,* PRF1*,* NRP1*,* IL19*,* TMEM161A*,***FASLG***,* PMAIP1*,* AURKB*,* SFN*,* CDKN2A*,* MYOCD*,***IFNG***,* CHST11*,* ROBO4*,* VNN1*,* MX1*,* SRGN*,* PHLDA1*,* EGR1*,* ZFP36*,* ST6GAL1*,* IL2RB*,* VAV3*,* MAGI1*,* AIMP1*,* SOCS3*,***GZMA***,* BCL2A1*,* PIM1*,* NR4A1*,***GZMB***,* NR4A3*,* DOCK8*,* ANXA4*,* PLAC8*,* SOD2*,* PLAUR*,* PRKCB*,* CD38*,* CORO1A*,* ADM*,* SIX1*,* LGALS14*,* TNFAIP8*,* HIF3A*,* EMP1*,* HTR2A*,* DUSP6*,* PRODH*
GOTERM_BP_FAT	Regulation of T cell differentiation in thymus	5	5.38E‐04	*CDKN2A*,* CAMK4*,* RASGRP1*,* VNN1*,* ZEB1*
GOTERM_BP_FAT	Regulation of thymocyte aggregation	5	5.38E‐04	*CDKN2A*,* CAMK4*,* RASGRP1*,* VNN1*,* ZEB1*
GOTERM_BP_FAT	Positive regulation of chemotaxis	9	5.69E‐04	*FGF18*,* NRP1*,* RAC2*,* CXCL13*,* CCR1*,* CXCL2*,* LBP*,* XCL1*,* CCL4*
GOTERM_BP_FAT	Regulation of chemotaxis	11	5.84E‐04	*FGF18*,* ST6GAL1*,* NRP1*,* RAC2*,* CXCL13*,* CCR1*,* CXCL2*,* LBP*,* XCL1*,* ANGPT2*,* CCL4*
GOTERM_BP_FAT	Neutrophil migration	8	5.86E‐04	*VAV3*,* RAC2*,* CXCL2*,***IFNG***,* TREM1*,* LBP*,* XCL1*,* CCL4*
GOTERM_BP_FAT	Humoral immune response	12	6.16E‐04	*CD55*,* ST6GAL1*,* ADM*,* CFB*,* RNASE7*,***IFNG***,* C1RL*,* CFI*,* C1S*,* TREM1*,* TRDC*,* BLNK*
GOTERM_BP_FAT	Leukocyte chemotaxis	11	6.34E‐04	*CORO1A*,* VAV3*,* RAC2*,* CXCL13*,* CCR1*,* CXCL2*,***IFNG***,* TREM1*,* LBP*,* XCL1*,* CCL4*
GOTERM_BP_FAT	Regulation of cell death	40	6.46E‐04	*FGFR2*,* NRP1*,* IL19*,* TMEM161A*,***FASLG***,* AURKB*,* SFN*,* PMAIP1*,* CDKN2A*,* MYOCD*,***IFNG***,* CHST11*,* ROBO4*,* VNN1*,* PHLDA1*,* ZFP36*,* EGR1*,* ST6GAL1*,* IL2RB*,* VAV3*,* SOCS3*,***GZMA***,* BCL2A1*,* PIM1*,* NR4A1*,***GZMB***,* DOCK8*,* NR4A3*,* ANXA4*,* PLAUR*,* PLAC8*,* SOD2*,* CD38*,* CORO1A*,* ADM*,* SIX1*,* LGALS14*,* TNFAIP8*,* DUSP6*,* PRODH*
GOTERM_BP_FAT	Negative regulation of apoptotic process	26	6.57E‐04	*FGFR2*,* NRP1*,* IL19*,* TMEM161A*,***FASLG***,* AURKB*,* SFN*,* MYOCD*,* CHST11*,* VNN1*,* ST6GAL1*,* IL2RB*,* SOCS3*,* BCL2A1*,* PIM1*,* NR4A1*,* NR4A3*,* DOCK8*,* ANXA4*,* SOD2*,* PLAC8*,* PLAUR*,* CD38*,* CORO1A*,* SIX1*,* TNFAIP8*
GOTERM_BP_FAT	Innate immune response	26	6.99E‐04	*KLRC2*,* ASS1*,* TRDC*,* C1S*,* CCL4*,* CD96*,* IRAK3*,* RASGRP1*,* RNASE7*,***IFNG***,* VNN1*,* CFI*,* LBP*,* MX1*,* KLRD1*,* EGR1*,* CFB*,* SOCS3*,***GZMB***,* CD84*,* CORO1A*,* CD55*,* C1RL*,* TREM1*,* XCL1*,* KIR3DL1*
GOTERM_BP_FAT	Glycosaminoglycan metabolic process	10	7.21E‐04	*ARSB*,* CHSY3*,* HPSE*,* CHST6*,* B3GNT7*,* CHST11*,* PIM1*,* HSPG2*,* VCAN*,* HS2ST1*
GOTERM_BP_FAT	Programmed cell death	46	7.29E‐04	*FGFR2*,* PRF1*,* NRP1*,* IL19*,* TMEM161A*,***FASLG***,* AURKB*,* SFN*,* PMAIP1*,* CDKN2A*,* MYOCD*,***IFNG***,* CHST11*,* ROBO4*,* VNN1*,* MX1*,* SRGN*,* PHLDA1*,* ZFP36*,* EGR1*,* ST6GAL1*,* IL2RB*,* VAV3*,* AIMP1*,* SOCS3*,***GZMA***,* BCL2A1*,* PIM1*,* NR4A1*,***GZMB***,* DOCK8*,* NR4A3*,* ANXA4*,* PRKCB*,* PLAUR*,* PLAC8*,* SOD2*,* CD38*,* CORO1A*,* ADM*,* SIX1*,* LGALS14*,* TNFAIP8*,* HIF3A*,* DUSP6*,* PRODH*
GOTERM_BP_FAT	Apoptotic process	44	7.55E‐04	*FGFR2*,* PRF1*,* NRP1*,* IL19*,* TMEM161A*,***FASLG***,* AURKB*,* SFN*,* PMAIP1*,* CDKN2A*,* MYOCD*,***IFNG***,* CHST11*,* VNN1*,* MX1*,* SRGN*,* PHLDA1*,* ZFP36*,* ST6GAL1*,* IL2RB*,* VAV3*,* AIMP1*,* SOCS3*,***GZMA***,* BCL2A1*,* PIM1*,* NR4A1*,***GZMB***,* DOCK8*,* NR4A3*,* ANXA4*,* PRKCB*,* PLAUR*,* PLAC8*,* SOD2*,* CD38*,* CORO1A*,* ADM*,* SIX1*,* LGALS14*,* TNFAIP8*,* HIF3A*,* DUSP6*,* PRODH*
GOTERM_BP_FAT	Negative regulation of programmed cell death	26	7.88E‐04	*FGFR2*,* NRP1*,* IL19*,* TMEM161A*,***FASLG***,* AURKB*,* SFN*,* MYOCD*,* CHST11*,* VNN1*,* ST6GAL1*,* IL2RB*,* SOCS3*,* BCL2A1*,* PIM1*,* NR4A1*,* NR4A3*,* DOCK8*,* ANXA4*,* SOD2*,* PLAC8*,* PLAUR*,* CD38*,* CORO1A*,* SIX1*,* TNFAIP8*
GOTERM_BP_FAT	Granulocyte chemotaxis	8	8.39E‐04	*VAV3*,* RAC2*,* CXCL2*,***IFNG***,* TREM1*,* LBP*,* XCL1*,* CCL4*
GOTERM_BP_FAT	Cytokine production involved in immune response	7	8.41E‐04	*IRAK3*,* CD96*,* CD55*,* CAMK4*,* NR4A3*,* TREM1*,* XCL1*
GOTERM_BP_FAT	Positive regulation of immune system process	28	8.58E‐04	*RBP4*,* PTPRC*,* VAV3*,* CFB*,* CCR1*,* CXCL2*,* C1S*,* TRDC*,* NR4A3*,* CCL4*,* PRKCB*,* CD84*,* FOS*,* IRAK3*,* CD38*,* CD55*,* MYO10*,* CORO1A*,* RAC2*,* CXCL13*,* RASGRP1*,***IFNG***,* C1RL*,* VNN1*,* LBP*,* CFI*,* XCL1*,* DPP4*
GOTERM_BP_FAT	Leukocyte apoptotic process	8	8.88E‐04	*ST6GAL1*,* CDKN2A*,* LGALS14*,***IFNG***,***FASLG***,* NR4A3*,* DOCK8*,* AURKB*
GOTERM_BP_FAT	Production of molecular mediator of immune response	10	.001012913	*PTPRC*,* IRAK3*,* CD96*,* RBP4*,* CD55*,* CAMK4*,***IFNG***,* NR4A3*,* TREM1*,* XCL1*
GOTERM_BP_FAT	Regulation of mononuclear cell proliferation	11	.001040266	*PTPRC*,* CD38*,* CD55*,* ST6GAL1*,* CORO1A*,* VAV3*,* CDKN2A*,* RAC2*,* IL20RB*,***IFNG***,* XCL1*
GOTERM_BP_FAT	Aminoglycan metabolic process	10	.001061591	*ARSB*,* CHSY3*,* HPSE*,* CHST6*,* B3GNT7*,* CHST11*,* PIM1*,* HSPG2*,* VCAN*,* HS2ST1*
GOTERM_BP_FAT	Response to bacterium	20	.001085717	*ZFP36*,* ASS1*,* SOCS3*,* GNLY*,* CXCL2*,* STATH*,***FASLG***,* TRDC*,* SOD2*,* PLAC8*,* CD96*,* IRAK3*,* FOS*,* THBD*,* ADM*,* CXCL13*,* RNASE7*,***IFNG***,* TREM1*,* LBP*
GOTERM_BP_FAT	Leukocyte cell–cell adhesion	18	.001187931	*EGR1*,* PTPRC*,* ASS1*,* LEPR*,* NR4A3*,* ZEB1*,* DOCK8*,* CORO1A*,* CD55*,* CDKN2A*,* RAC2*,* CAMK4*,* IL20RB*,* RASGRP1*,***IFNG***,* VNN1*,* XCL1*,* DPP4*
GOTERM_MF_FAT	Glycosaminoglycan binding	11	.001232209	*FGFR2*,* F11*,***TNFAIP6***,* NRP1*,* CXCL13*,* RNASE7*,* MMP7*,* COL25A1*,* VCAN*,* THBS2*,* HABP2*
GOTERM_BP_FAT	Leukocyte aggregation	17	.001364809	*EGR1*,* PTPRC*,* LEPR*,* NR4A3*,* ZEB1*,* DOCK8*,* CORO1A*,* CD55*,* CDKN2A*,* RAC2*,* CAMK4*,* IL20RB*,* RASGRP1*,***IFNG***,* VNN1*,* XCL1*,* DPP4*
GOTERM_BP_FAT	Regulation of lymphocyte activation	16	.001403667	*PTPRC*,* VAV3*,* TRDC*,* ZEB1*,* CD38*,* CORO1A*,* CD55*,* CDKN2A*,* RAC2*,* CAMK4*,* IL20RB*,* RASGRP1*,***IFNG***,* VNN1*,* XCL1*,* DPP4*
GOTERM_BP_FAT	Regulation of leukocyte proliferation	11	.00143279	*PTPRC*,* CD38*,* CD55*,* ST6GAL1*,* CORO1A*,* VAV3*,* CDKN2A*,* RAC2*,* IL20RB*,***IFNG***,* XCL1*
GOTERM_BP_FAT	Leukocyte mediated cytotoxicity	7	.001521044	*PTPRC*,* CORO1A*,* RASGRP1*,***GZMB***,* TREM1*,* XCL1*,* KIR3DL1*
GOTERM_BP_FAT	Positive regulation of leukocyte chemotaxis	7	.001521044	*RAC2*,* CXCL13*,* CCR1*,* CXCL2*,* LBP*,* XCL1*,* CCL4*
GOTERM_BP_FAT	Response to lipopolysaccharide	14	.001525141	*ZFP36*,* IRAK3*,* CD96*,* FOS*,* THBD*,* ADM*,* ASS1*,* SOCS3*,* CXCL13*,* CXCL2*,***IFNG***,***FASLG***,* LBP*,* SOD2*
GOTERM_BP_FAT	Granulocyte migration	8	.001597708	*VAV3*,* RAC2*,* CXCL2*,***IFNG***,* TREM1*,* LBP*,* XCL1*,* CCL4*
GOTERM_BP_FAT	Response to lipid	27	.001721042	*RBP4*,* THRB*,* ASS1*,* RBP1*,* CXCL2*,* TMEM161A*,***FASLG***,* CD96*,* IRAK3*,* FOS*,***IFNG***,* LBP*,* NR2F1*,* ZFP36*,* SOCS3*,* PIM1*,* NR4A1*,* FOSB*,* NR4A3*,* SOD2*,* CD38*,* SSTR2*,* THBD*,* DKK1*,* ADM*,* CXCL13*,* FABP3*
GOTERM_BP_FAT	Response to drug	17	.001755254	*SLC8A1*,* VAV3*,* ASS1*,* CYP2B6*,* CYP2C9*,* SOCS3*,* FOSB*,* CCL4*,* SOD2*,* CD38*,* FOS*,* APOD*,* CD69*,***IFNG***,* FABP3*,* HTR2A*,* DUSP6*
GOTERM_BP_FAT	Regulation of cell adhesion	21	.001839233	*PTPRC*,* ST6GAL1*,* VAV3*,* ASS1*,* ZEB1*,* NR4A3*,* CORO1A*,* CD55*,* MYO10*,* CDKN2A*,* RAC2*,* CAMK4*,* IL20RB*,* APOD*,* CXCL13*,* RASGRP1*,***IFNG***,* VNN1*,* XCL1*,* ANGPT2*,* DPP4*
GOTERM_BP_FAT	Regulation of T cell activation	13	.001997259	*PTPRC*,* ZEB1*,* CORO1A*,* CD55*,* CDKN2A*,* RAC2*,* IL20RB*,* CAMK4*,* RASGRP1*,***IFNG***,* VNN1*,* XCL1*,* DPP4*
GOTERM_BP_FAT	Negative regulation of cytokine production	11	.002001253	*CD84*,* ZFP36*,* IRAK3*,* CD96*,* APOD*,* IL20RB*,***IFNG***,* LBP*,* XCL1*,* ANXA4*,* SRGN*
GOTERM_BP_FAT	Cellular defense response	6	.002050165	*PRF1*,* KLRC2*,* GNLY*,* LBP*,* KIR2DL4*,* KIR3DL2*
GOTERM_BP_FAT	Inflammatory cell apoptotic process	4	.002205885	*ST6GAL1*,* CDKN2A*,***IFNG***,***FASLG***
GOTERM_BP_FAT	Response to molecule of bacterial origin	14	.002213254	*ZFP36*,* IRAK3*,* CD96*,* FOS*,* THBD*,* ADM*,* ASS1*,* SOCS3*,* CXCL13*,* CXCL2*,***IFNG***,***FASLG***,* LBP*,* SOD2*
GOTERM_BP_FAT	Negative regulation of cell death	26	.00245476	*FGFR2*,* NRP1*,* IL19*,* TMEM161A*,***FASLG***,* AURKB*,* SFN*,* MYOCD*,* CHST11*,* VNN1*,* ST6GAL1*,* IL2RB*,* SOCS3*,* BCL2A1*,* PIM1*,* NR4A1*,* NR4A3*,* DOCK8*,* ANXA4*,* SOD2*,* PLAC8*,* PLAUR*,* CD38*,* CORO1A*,* SIX1*,* TNFAIP8*
GOTERM_BP_FAT	Mononuclear cell proliferation	12	.002460795	*PTPRC*,* CD38*,* CD55*,* ST6GAL1*,* CORO1A*,* VAV3*,* CDKN2A*,* RAC2*,* IL20RB*,***IFNG***,* DOCK8*,* XCL1*
GOTERM_BP_FAT	Response to wounding	21	.002508224	*ZFP36*,* F11*,* NRP1*,* VAV3*,* AIMP1*,* CCR1*,* DOCK8*,* SLC7A11*,* PRKCB*,* PLAUR*,* SOD2*,* THBD*,* RAC2*,* APOD*,* ADM*,* HPSE*,* INPP5F*,* H3F3B*,* IGFBP1*,* PAPSS2*,* FABP5*
GOTERM_BP_FAT	Positive regulation of cell proliferation	25	.002576078	*FGFR2*,* FGF18*,* NRP1*,***FASLG***,* RAC2*,* MYOCD*,* HPSE*,***IFNG***,* CALCRL*,* DPP4*,* EGR1*,* PTPRC*,* ST6GAL1*,* VAV3*,* NR4A1*,* NR4A3*,* PLAC8*,* CD38*,* CORO1A*,* CD55*,* ADM*,* SIX1*,* ID4*,* XCL1*,* HTR2A*
GOTERM_BP_FAT	Myeloid leukocyte migration	9	.002633977	*VAV3*,* RAC2*,* CCR1*,* CXCL2*,***IFNG***,* TREM1*,* LBP*,* XCL1*,* CCL4*
GOTERM_BP_FAT	Positive regulation of leukocyte activation	13	.002679897	*PTPRC*,* CD38*,* CD55*,* CORO1A*,* VAV3*,* RASGRP1*,***IFNG***,* VNN1*,* LBP*,* NR4A3*,* TRDC*,* XCL1*,* DPP4*
GOTERM_BP_FAT	Response to extracellular stimulus	17	.002734588	*ZFP36*,* ARSB*,* SLC8A1*,* ASS1*,* RBP1*,* SOCS3*,* PIM1*,* MMP7*,* GAST*,* PMAIP1*,* SOD2*,* FOS*,* SSTR2*,* MYOCD*,* ADM*,* SLC2A1*,* CP*
GOTERM_BP_FAT	Multi–multicellular organism process	11	.002740849	*CD38*,* FOS*,* CD55*,* PSG9*,* THBD*,* ADM*,* MMP7*,* H3F3B*,* SLC38A1*,* FOSB*,* ANGPT2*
GOTERM_BP_FAT	Regulation of inflammatory response	13	.002812705	*ZFP36*,***TNFAIP6***,* CD55*,* APOD*,* IL20RB*,* SOCS3*,* CFB*,* SLC7A2*,* CFI*,* CALCRL*,* LBP*,* XCL1*,* CCL4*
GOTERM_BP_FAT	Single organismal cell–cell adhesion	22	.002886539	*EGR1*,* PTPRC*,* ASS1*,* LEPR*,* DOCK8*,* NR4A3*,* ZEB1*,* SLC7A11*,* NPHP4*,* CORO1A*,* CD55*,* MYO10*,* CDKN2A*,* RAC2*,* CAMK4*,* IL20RB*,* CXCL13*,* RASGRP1*,***IFNG***,* VNN1*,* XCL1*,* DPP4*
GOTERM_CC_FAT	Cell surface	24	.002962228	*PPFIA2*,* ARSB*,* FGFR2*,* PTPRC*,* IL2RB*,* NRP1*,* TRPM8*,* AIMP1*,* CCR1*,* MMP7*,***FASLG***,* TRDC*,* ANXA4*,* SLC7A11*,* PROM1*,* CD38*,* CD55*,* THBD*,* CD69*,***IFNG***,* LBP*,* DPP6*,* KLRD1*,* DPP4*
GOTERM_BP_FAT	T cell aggregation	16	.002988034	*EGR1*,* PTPRC*,* LEPR*,* ZEB1*,* DOCK8*,* CORO1A*,* CD55*,* CDKN2A*,* RAC2*,* CAMK4*,* IL20RB*,* RASGRP1*,***IFNG***,* VNN1*,* XCL1*,* DPP4*
GOTERM_BP_FAT	T cell activation	16	.002988034	*EGR1*,* PTPRC*,* LEPR*,* ZEB1*,* DOCK8*,* CORO1A*,* CD55*,* CDKN2A*,* RAC2*,* CAMK4*,* IL20RB*,* RASGRP1*,***IFNG***,* VNN1*,* XCL1*,* DPP4*
GOTERM_BP_FAT	Lymphocyte aggregation	16	.003046718	*EGR1*,* PTPRC*,* LEPR*,* ZEB1*,* DOCK8*,* CORO1A*,* CD55*,* CDKN2A*,* RAC2*,* CAMK4*,* IL20RB*,* RASGRP1*,***IFNG***,* VNN1*,* XCL1*,* DPP4*
GOTERM_BP_FAT	Cellular modified amino acid metabolic process	9	.003065973	*CHDH*,* MTHFD2*,* MTHFS*,* GSTT2B*,* ASS1*,* SLCO4A1*,* MTHFD1L*,* PRODH*,* SOD2*
GOTERM_BP_FAT	Negative regulation of protein serine/threonine kinase activity	8	.003199245	*DUSP5*,* IRAK3*,* CDKN2A*,* MYOCD*,* PKIB*,* SFN*,* RGS14*,* DUSP6*
GOTERM_BP_FAT	Positive regulation of cell migration	15	.003220579	*PTPRC*,***TNFAIP6***,* FGF18*,* SLC8A1*,* CORO1A*,* NRP1*,* RAC2*,* CXCL13*,* CCR1*,* CXCL2*,***IFNG***,* DOCK7*,* LBP*,* XCL1*,* CCL4*
GOTERM_BP_FAT	Aging	13	.003269145	*ASS1*,* SOCS3*,* BCL2A1*,* MMP7*,* AURKB*,* SOD2*,* FOS*,* CDKN2A*,* APOD*,* ADM*,* IGFBP1*,* CP*,* HTR2A*
GOTERM_BP_FAT	Positive regulation of cell activation	13	.003376692	*PTPRC*,* CD38*,* CD55*,* CORO1A*,* VAV3*,* RASGRP1*,***IFNG***,* VNN1*,* LBP*,* NR4A3*,* TRDC*,* XCL1*,* DPP4*
GOTERM_BP_FAT	Female pregnancy	10	.003499079	*CD38*,* FOS*,* PSG9*,* THBD*,* ADM*,* MMP7*,* H3F3B*,* SLC38A1*,* FOSB*,* ANGPT2*
GOTERM_BP_FAT	Regulation of lymphocyte proliferation	10	.003499079	*PTPRC*,* CD38*,* CD55*,* CORO1A*,* VAV3*,* CDKN2A*,* RAC2*,* IL20RB*,***IFNG***,* XCL1*
GOTERM_BP_FAT	Leukocyte proliferation	12	.003703152	*PTPRC*,* CD38*,* CD55*,* ST6GAL1*,* CORO1A*,* VAV3*,* CDKN2A*,* RAC2*,* IL20RB*,***IFNG***,* DOCK8*,* XCL1*
GOTERM_BP_FAT	Defense response to other organism	19	.003727248	*PRF1*,* PTPRC*,* DPF3*,* AIMP1*,* GNLY*,* STATH*,* LMCD1*,* TRDC*,* PMAIP1*,* COTL1*,* PLAC8*,* ADM*,* CXCL13*,* RNASE7*,***IFNG***,* TREM1*,* LBP*,* MX1*,* FCGR3B*
GOTERM_BP_FAT	Regulation of leukocyte chemotaxis	7	.004046973	*RAC2*,* CXCL13*,* CCR1*,* CXCL2*,* LBP*,* XCL1*,* CCL4*
GOTERM_BP_FAT	Negative regulation of immune effector process	7	.004245428	*CD84*,* PTPRC*,* IRAK3*,* CD96*,* CD55*,* IL20RB*,* XCL1*
GOTERM_BP_FAT	Positive regulation of mononuclear cell proliferation	8	.00427214	*PTPRC*,* CD38*,* CD55*,* ST6GAL1*,* CORO1A*,* VAV3*,***IFNG***,* XCL1*
GOTERM_BP_FAT	Positive regulation of cell motility	15	.004330368	*PTPRC*,***TNFAIP6***,* FGF18*,* SLC8A1*,* CORO1A*,* NRP1*,* RAC2*,* CXCL13*,* CCR1*,* CXCL2*,***IFNG***,* DOCK7*,* LBP*,* XCL1*,* CCL4*
GOTERM_BP_FAT	Positive regulation of cell–cell adhesion	11	.004408001	*PTPRC*,* CD55*,* MYO10*,* CORO1A*,* CXCL13*,* RASGRP1*,***IFNG***,* VNN1*,* NR4A3*,* XCL1*,* DPP4*
GOTERM_BP_FAT	Response to cold	5	.00466463	*FOS*,* ADM*,* TRPM8*,* PLAC8*,* SOD2*
GOTERM_BP_FAT	Positive regulation of neutrophil chemotaxis	4	.005125974	*RAC2*,* CXCL2*,* LBP*,* XCL1*
GOTERM_MF_FAT	Aminopeptidase activity	5	.005195241	*F11*,* METAP1D*,* PHEX*,* DPP6*,* DPP4*
GOTERM_BP_FAT	Positive regulation of leukocyte proliferation	8	.00539139	*PTPRC*,* CD38*,* CD55*,* ST6GAL1*,* CORO1A*,* VAV3*,***IFNG***,* XCL1*
GOTERM_BP_FAT	Negative regulation of leukocyte mediated immunity	5	.005435985	*CD84*,* PTPRC*,* CD96*,* IL20RB*,* XCL1*
GOTERM_BP_FAT	Positive regulation of cellular component movement	15	.005527572	*PTPRC*,***TNFAIP6***,* FGF18*,* SLC8A1*,* CORO1A*,* NRP1*,* RAC2*,* CXCL13*,* CCR1*,* CXCL2*,***IFNG***,* DOCK7*,* LBP*,* XCL1*,* CCL4*
GOTERM_BP_FAT	Positive regulation of locomotion	15	.005622113	*PTPRC*,***TNFAIP6***,* FGF18*,* SLC8A1*,* CORO1A*,* NRP1*,* RAC2*,* CXCL13*,* CCR1*,* CXCL2*,***IFNG***,* DOCK7*,* LBP*,* XCL1*,* CCL4*
GOTERM_BP_FAT	Endothelial cell chemotaxis	4	.005762154	*FGF18*,* NRP1*,* CXCL13*,* NR4A1*
GOTERM_BP_FAT	Myeloid leukocyte mediated immunity	6	.005944619	*CD84*,* RAC2*,* CAMK4*,* RASGRP1*,* NR4A3*,* TREM1*
GOTERM_BP_FAT	Renal system vasculature development	4	.006443547	*EGR1*,* NRP1*,***IFNG***,* ANGPT2*
GOTERM_BP_FAT	Kidney vasculature development	4	.006443547	*EGR1*,* NRP1*,***IFNG***,* ANGPT2*
GOTERM_BP_FAT	Single organism cell adhesion	22	.006492015	*EGR1*,* PTPRC*,* ASS1*,* LEPR*,* DOCK8*,* NR4A3*,* ZEB1*,* SLC7A11*,* NPHP4*,* CORO1A*,* CD55*,* MYO10*,* CDKN2A*,* RAC2*,* CAMK4*,* IL20RB*,* CXCL13*,* RASGRP1*,***IFNG***,* VNN1*,* XCL1*,* DPP4*
GOTERM_BP_FAT	Regulation of locomotion	22	.006605694	*ARSB*,* PTPRC*,* FGF18*,* SLC8A1*,* ST6GAL1*,* NRP1*,* CCR1*,* CXCL2*,* DOCK7*,* CCL4*,***TNFAIP6***,* MMP10*,* CORO1A*,* RAC2*,* APOD*,* CXCL13*,***IFNG***,* ROBO4*,* INPP5F*,* LBP*,* XCL1*,* ANGPT2*
GOTERM_BP_FAT	Glycosaminoglycan biosynthetic process	7	.006645161	*CHSY3*,* CHST6*,* B3GNT7*,* CHST11*,* HSPG2*,* VCAN*,* HS2ST1*
GOTERM_BP_FAT	Cytokine production	19	.006800751	*ZFP36*,* EGR1*,* IL19*,* NR4A3*,* ANXA4*,* CD84*,* CD96*,* IRAK3*,* CD55*,* CAMK4*,* IL20RB*,* APOD*,* HPSE*,* RASGRP1*,***IFNG***,* TREM1*,* LBP*,* XCL1*,* SRGN*
GOTERM_BP_FAT	Lymphocyte proliferation	11	.006835525	*PTPRC*,* CD38*,* CD55*,* CORO1A*,* VAV3*,* CDKN2A*,* RAC2*,* IL20RB*,***IFNG***,* DOCK8*,* XCL1*
GOTERM_BP_FAT	Aminoglycan biosynthetic process	7	.006929937	*CHSY3*,* CHST6*,* B3GNT7*,* CHST11*,* HSPG2*,* VCAN*,* HS2ST1*
GOTERM_BP_FAT	Mucopolysaccharide metabolic process	7	.006929937	*ARSB*,* CHSY3*,* CHST6*,* B3GNT7*,* CHST11*,* PIM1*,* VCAN*
GOTERM_BP_FAT	Cellular response to chemical stimulus	56	.007101409	*FGF18*,* NRP1*,* THRB*,* LEPR*,* TMEM161A*,***FASLG***,* PMAIP1*,* ZEB1*,* FOS*,* MYOCD*,* CHST11*,***IFNG***,* VNN1*,* CALCRL*,* LBP*,* MX1*,* ANGPT2*,* CSF2RA*,* NR2F1*,* ZFP36*,* EGR1*,* SATB2*,* SOCS3*,* PIM1*,* FOSB*,* PRKCB*,* SSTR2*,* IL20RB*,* GUCY1B3*,* TREM1*,* PRODH*,* FGFR2*,* ASS1*,* CYP2B6*,* CCR1*,* CXCL2*,* CCL4*,* IRAK3*,* RAC2*,* CD69*,* UGT1A5*,* PTPRC*,* IL2RB*,* SLC8A1*,* VAV3*,* CYP2C9*,* NR4A1*,* NR4A3*,* SOD2*,* CORO1A*,* DKK1*,* CXCL13*,* HIF3A*,* IGFBP1*,* XCL1*,* DUSP6*
GOTERM_BP_FAT	Positive regulation of granulocyte chemotaxis	4	.007170933	*RAC2*,* CXCL2*,* LBP*,* XCL1*
GOTERM_BP_FAT	Lymphocyte differentiation	12	.007197221	*EGR1*,* PTPRC*,* CDKN2A*,* CAMK4*,* IKZF1*,* LEPR*,* RASGRP1*,***IFNG***,* CMTM7*,* VNN1*,* ZEB1*,* BLNK*
GOTERM_BP_FAT	Epithelial cell migration	10	.007524598	*ARSB*,* FGF18*,* NRP1*,* S100P*,* CXCL13*,***IFNG***,* FERMT1*,* NR4A1*,* ANGPT2*,* DPP4*
GOTERM_BP_FAT	Positive regulation of leukocyte migration	7	.00752586	*RAC2*,* CXCL13*,* CCR1*,* CXCL2*,* LBP*,* XCL1*,* CCL4*
GOTERM_BP_FAT	Regulation of neutrophil chemotaxis	4	.007945027	*RAC2*,* CXCL2*,* LBP*,* XCL1*
GOTERM_BP_FAT	Epithelium migration	10	.008151142	*ARSB*,* FGF18*,* NRP1*,* S100P*,* CXCL13*,***IFNG***,* FERMT1*,* NR4A1*,* ANGPT2*,* DPP4*
GOTERM_BP_FAT	Formation of primary germ layer	7	.008157921	*FGFR2*,* DUSP5*,* LAMB3*,* DKK1*,* ETS2*,* NR4A3*,* DUSP6*
GOTERM_BP_FAT	Response to hypoxia	12	.008231471	*EGR1*,* CD38*,* SLC8A1*,* ADM*,* MYOCD*,* SOCS3*,* HIF3A*,* PMAIP1*,* ANGPT2*,* DPP4*,* PRKCB*,* SOD2*
GOTERM_BP_FAT	Regulation of cell migration	20	.008409748	*ARSB*,* PTPRC*,* FGF18*,* SLC8A1*,* NRP1*,* CCR1*,* CXCL2*,* DOCK7*,* CCL4*,***TNFAIP6***,* MMP10*,* CORO1A*,* RAC2*,* APOD*,* CXCL13*,***IFNG***,* ROBO4*,* LBP*,* XCL1*,* ANGPT2*
GOTERM_BP_FAT	Regulation of cell motility	21	.008458009	*ARSB*,* PTPRC*,* FGF18*,* SLC8A1*,* NRP1*,* CCR1*,* CXCL2*,* DOCK7*,* CCL4*,***TNFAIP6***,* MMP10*,* CORO1A*,* RAC2*,* APOD*,* CXCL13*,***IFNG***,* ROBO4*,* INPP5F*,* LBP*,* XCL1*,* ANGPT2*
GOTERM_BP_FAT	Regulation of leukocyte apoptotic process	6	.008502574	*ST6GAL1*,* CDKN2A*,* LGALS14*,* NR4A3*,* DOCK8*,* AURKB*
GOTERM_BP_FAT	Regulation of response to stress	33	.008516958	*DPF3*,* TMEM161A*,* PMAIP1*,* CCL4*,* CD96*,* IRAK3*,* CDKN2A*,* APOD*,* HPSE*,* RASGRP1*,***IFNG***,* INPP5F*,* VNN1*,* LBP*,* CFI*,* CALCRL*,* FCGR3B*,* ZFP36*,* F11*,* SOCS3*,* CFB*,* LMCD1*,* NR4A3*,* PLAUR*,* SOD2*,***TNFAIP6***,* CD55*,* THBD*,* IL20RB*,* HELB*,* SLC7A2*,* TREM1*,* XCL1*
GOTERM_BP_FAT	Negative regulation of defense response	8	.008555067	*ZFP36*,* IRAK3*,* CD96*,***TNFAIP6***,* APOD*,* IL20RB*,* SOCS3*,* CALCRL*
GOTERM_BP_FAT	Ameboidal‐type cell migration	12	.008776187	*ARSB*,* ZFAND5*,* FGF18*,* SLC8A1*,* NRP1*,* S100P*,* CXCL13*,***IFNG***,* FERMT1*,* NR4A1*,* ANGPT2*,* DPP4*
GOTERM_BP_FAT	Response to oxygen‐containing compound	36	.008811937	*RBP4*,* ASS1*,* CXCL2*,* TMEM161A*,***FASLG***,* ZEB1*,* FOS*,* CD96*,* IRAK3*,* APOD*,***IFNG***,* LBP*,* CALCRL*,* ANGPT2*,* EGR1*,* ZFP36*,* SLC8A1*,* ST6GAL1*,* SOCS3*,* PIM1*,* NR4A1*,* FOSB*,* NR4A3*,* PRKCB*,* SOD2*,* CD38*,* SSTR2*,* CD55*,* DKK1*,* THBD*,* ADM*,* CXCL13*,* FABP3*,* GUCY1B3*,* IGFBP1*,* HTR2A*
GOTERM_BP_FAT	Response to nutrient levels	15	.008839745	*ZFP36*,* ARSB*,* SLC8A1*,* ASS1*,* RBP1*,* SOCS3*,* PIM1*,* MMP7*,* GAST*,* PMAIP1*,* SOD2*,* SSTR2*,* ADM*,* SLC2A1*,* CP*
GOTERM_BP_FAT	Regulation of T cell proliferation	8	.008844981	*PTPRC*,* CD55*,* CORO1A*,* CDKN2A*,* RAC2*,* IL20RB*,***IFNG***,* XCL1*
GOTERM_BP_FAT	Negative regulation of transcription from RNA polymerase II promoter	21	.00904159	*ZFP36*,* EGR1*,* FGFR2*,* SATB2*,* THRB*,* IKZF1*,* LMCD1*,***FASLG***,* FOSB*,* AURKB*,* NR4A3*,* ZEB1*,* DKK1*,* MYOCD*,* ETS2*,* SIX1*,***IFNG***,* ID4*,* BHLHE40*,* S100A1*,* NR2F1*
GOTERM_BP_FAT	Apoptotic mitochondrial changes	7	.009176322	*CDKN2A*,* BCL2A1*,***GZMB***,* PMAIP1*,* SFN*,* SOD2*,* PLAUR*
GOTERM_BP_FAT	Regulation of leukocyte mediated immunity	8	.009445487	*CD84*,* PTPRC*,* CD96*,* RAC2*,* IL20RB*,* RASGRP1*,***IFNG***,* XCL1*
GOTERM_BP_FAT	Tissue migration	10	.009536961	*ARSB*,* FGF18*,* NRP1*,* S100P*,* CXCL13*,***IFNG***,* FERMT1*,* NR4A1*,* ANGPT2*,* DPP4*
GOTERM_BP_FAT	Positive regulation of neutrophil migration	4	.009635883	*RAC2*,* CXCL2*,* LBP*,* XCL1*
GOTERM_BP_FAT	Urogenital system development	12	.00975718	*FGFR2*,* PROM1*,* EGR1*,* RBP4*,* NRP1*,* ASS1*,* MYOCD*,* SERPINB5*,* SIX1*,***IFNG***,* ID4*,* ANGPT2*
GOTERM_BP_FAT	Negative regulation of protein kinase activity	10	.009782435	*DUSP5*,* PTPRC*,* IRAK3*,* CDKN2A*,* MYOCD*,* SOCS3*,* PKIB*,* SFN*,* RGS14*,* DUSP6*
GOTERM_BP_FAT	Extracellular matrix disassembly	6	.009801526	*MMP10*,* LAMB3*,* HSPG2*,* MMP7*,* DPP4*,* MMP1*
GOTERM_BP_FAT	Fat cell differentiation	9	.00988103	*ZFP36*,* LAMB3*,* BBS9*,* NR4A1*,* ID4*,* NR4A3*,* PLAC8*,* HTR2A*,* SOD2*
GOTERM_BP_FAT	Embryonic skeletal system development	7	.009903808	*FGFR2*,* RBP4*,* SATB2*,* SIX1*,* CHST11*,* ZEB1*,* MTHFD1L*

Bold denotes gene names which are picked up in the results and discussion.

The KEGG pathways that were associated with these genes involved the pathways that were related to immunity, such as “natural killer cell mediated cytotoxicity,” “complement and coagulation cascades,” “antigen processing and presentation,” “Graft‐versus‐host disease,” and “allograft rejection” (Tables 3 and S2). These KEGG pathways also included *IFN‐*γ*, GZMB*, and *FASLG*.

**Table 3:** Kyoto Encyclopedia of Genes and Genomes pathway analysis for the genes that were up‐regulated in the thin endometrium

Term	Count	*P* value	Gene
Natural killer cell‐mediated cytotoxicity	10	8.21E‐04	*PRF1*,* VAV3*,* RAC2*,***IFNG***,***FASLG***,***GZMB***,* FCGR3B*,* KLRD1*,* KIR2DL4*,* PRKCB*
Complement and coagulation cascades	7	.00270751	*F11*,* CD55*,* THBD*,* CFB*,* CFI*,* C1S*,* PLAUR*
Transcriptional misregulation in cancer	10	.00722048	*PROM1*,* NFKBIZ*,* IL2RB*,* UTY*,* SIX1*,* BCL2A1*,* GRIA3*,***GZMB***,* H3F3B*,* NR4A3*
Antigen processing and presentation	6	.01912738	*KLRC2*,***IFNG***,* KLRD1*,* KIR3DL1*,* KIR2DL4*,* KIR3DL2*
Jak‐STAT signaling pathway	8	.02846128	*IL2RB*,* IL20RB*,* SOCS3*,* LEPR*,* IL19*,***IFNG***,* PIM1*,* CSF2RA*
Graft‐versus‐host disease	4	.02903814	*PRF1*,***IFNG***,***FASLG***,***GZMB***
Allograft rejection	4	.03900192	*PRF1*,***IFNG***,***FASLG***,***GZMB***
Amphetamine addiction	5	.04526534	*FOS*,* CAMK4*,* GRIA3*,* FOSB*,* PRKCB*
p53 signaling pathway	5	.04740558	*CDKN2A*,* SERPINB5*,* RRM2*,* PMAIP1*,* SFN*

Jak‐STAT, Janus kinase/signal transducers and activators of transcription. Bold denotes gene names which are picked up in the results and discussion.

### 3.3 Gene Ontology and Kyoto Encyclopedia of Genes and Genomes pathway analyses for the down‐regulated genes in the thin endometrium

The down‐regulated genes in the thin endometrium were related to metabolic processes, such as “small molecule catabolic process,” “single‐organism catabolic process,” “organic acid catabolic process,” and “carboxylic acid catabolic process” (Tables 4 and S3). The GO terms included genes for carnitine palmitoyltransferase I (*CPT1*), 3‐hydroxy‐3‐methylglutaryl‐coenzyme A (CoA) synthase 2 (*HMGCS2*), and 3‐oxoacid CoA‐transferase 1 (*OXCT1*), which are known to play important roles in generating energy in cells and tissues.^13^, ^14^, ^15^

**Table 4:** Gene ontology analysis for the genes that were down‐regulated in the thin endometrium

Category	Term	Count	*P* value	Gene
GOTERM_BP_FAT	Small molecule catabolic process	21	2.80E‐07	*NUDT16*,* ALDH6A1*,* ECI2*,* KYNU*,* SORD*,* BCKDHB*,* CYP26A1*,* ALDH3B2*,* HGD*,***CBR3***,* ACADL*,***CPT1A***,***OXCT1***,* FUT3*,* QPRT*,* GAD1*,* GPT2*,* PCCA*,* CROT*,* DCXR*,* XYLB*
GOTERM_BP_FAT	Single‐organism catabolic process	33	1.44E‐06	***XDH***,* KYNU*,* SORD*,***OXCT1***,* PDE1A*,* FUT3*,***IDH1***,* ENTPD3*,* PLCB1*,* GAD1*,* GPT2*,* NUDT16*,* ECI2*,* ALDH6A1*,* HERPUD1*,* PLD6*,* BCKDHB*,* HGD*,* ALDH3B2*,* CYP26A1*,* COL25A1*,***CBR3***,* COL5A3*,* ACADL*,***CPT1A***,* PLA2G4A*,* COL1A2*,* ACE2*,* QPRT*,* PCCA*,* DCXR*,* CROT*,* XYLB*
GOTERM_BP_FAT	Organic acid catabolic process	16	1.58E‐06	*ALDH6A1*,* ECI2*,* KYNU*,* SORD*,* BCKDHB*,* CYP26A1*,* HGD*,* ACADL*,***CPT1A***,* QPRT*,* GAD1*,* GPT2*,* PCCA*,* CROT*,* DCXR*,* XYLB*
GOTERM_BP_FAT	Carboxylic acid catabolic process	14	8.69E‐06	*ALDH6A1*,* ECI2*,* SORD*,* HGD*,* CYP26A1*,* ACADL*,***CPT1A***,* QPRT*,* GAD1*,* GPT2*,* PCCA*,* CROT*,* DCXR*,* XYLB*
GOTERM_BP_FAT	Oxidation‐reduction process	33	3.78E‐05	***XDH***,* TM7SF2*,* C15ORF48*,* PAM*,* STEAP4*,* CYP2J2*,* SORD*,* OPRK1*,***PPARG***,* DUOX1*,* FMO5*,***IDH1***,* NFATC4*,* SCD5*,* HHIP*,* ECI2*,* ALDH6A1*,* BCKDHB*,* HGD*,* ALDH3B2*,* CYP26A1*,* CYB5A*,***CBR3***,* ACADL*,***CPT1A***,* DHRS7*,* IYD*,* ACSM1*,* SQLE*,* GNAS*,* PHF8*,* CROT*,* DCXR*
GOTERM_BP_FAT	Secretion	36	4.37E‐05	***XDH***,* COPA*,* PAM*,* NAAA*,* OPRK1*,* PML*,* POSTN*,* TPD52*,* TLR6*,* TRH*,* KCNS3*,* CASP5*,* NOV*,* WNK4*,***OXCT1***,* SYN2*,* SYBU*,* CREB3L1*,* CHRNA6*,* GAD1*,* MAP2K6*,* ABCA12*,* TRPM4*,* ACTN1*,* GAL*,* ISL1*,* NLRP2*,* PCLO*,***CPT1A***,* PLA2G4A*,* CHGA*,* STXBP6*,* SYTL4*,* GNAS*,* CA2*,* CPB2*
GOTERM_BP_FAT	Carboxylic acid metabolic process	29	6.36E‐05	*PAM*,* KYNU*,* CYP2J2*,* SORD*,***PPARG***,* AGMAT*,***IDH1***,* UGT8*,* SCD5*,* GAD1*,* GPT2*,* GGTA1P*,* ECI2*,* ALDH6A1*,* PDK4*,* BCKDHB*,* HGD*,* CYP26A1*,* CYB5A*,* ACADL*,***CPT1A***,* ACSM3*,* ACSM1*,* PLA2G4A*,* QPRT*,* PCCA*,* DCXR*,* CROT*,* XYLB*
GOTERM_BP_FAT	Oxoacid metabolic process	29	7.06E‐05	*PAM*,* KYNU*,* CYP2J2*,* SORD*,***PPARG***,* AGMAT*,***IDH1***,* UGT8*,* SCD5*,* GAD1*,* GPT2*,* GGTA1P*,* ECI2*,* ALDH6A1*,* PDK4*,* BCKDHB*,* HGD*,* CYP26A1*,* CYB5A*,* ACADL*,***CPT1A***,* ACSM3*,* ACSM1*,* PLA2G4A*,* QPRT*,* PCCA*,* DCXR*,* CROT*,* XYLB*
GOTERM_BP_FAT	Peptide transport	15	9.83E‐05	*TRPM4*,* SLC15A2*,* ISL1*,* GAL*,* TRH*,* PCLO*,***CPT1A***,* KCNS3*,* NOV*,***OXCT1***,* TAP2*,* SYBU*,* SYTL4*,* GNAS*,* CA2*
GOTERM_BP_FAT	Transmembrane transport	39	1.02E‐04	*C15ORF48*,* CALHM1*,* SLC39A14*,* SLC38A4*,* ATP1B1*,* MFSD3*,* SLC15A2*,* OPRK1*,* KCNIP4*,* KCNS3*,* SLC24A4*,* MCOLN3*,* ANK3*,* TAP2*,* WNK4*,* SLC25A48*,* TTYH2*,* SLC39A8*,* CHRNA6*,* SLC43A1*,* ANO10*,* ABCA12*,* TRPM4*,* TRPM6*,* ABCC13*,* CYB5A*,* GAL*,* ANKH*,* ABCG1*,***CPT1A***,* GJB2*,* SLC26A3*,* ATP6V0E2*,* ADAMTS8*,* CLIC5*,* KCNN3*,* SLC7A1*,* CA2*,* SLC46A2*
GOTERM_BP_FAT	Ion transport	42	1.10E‐04	*C15ORF48*,* CALHM1*,* SLC39A14*,* STEAP4*,* SLC38A4*,* ATP1B1*,* MFSD3*,* SLC15A2*,* OPRK1*,***PPARG***,* PML*,* TRH*,* KCNIP4*,* SEC14L1*,* KCNS3*,* SLC24A4*,* MCOLN3*,* ANK3*,* WNK4*,* TTYH2*,* SLC39A8*,* CHRNA6*,* SLC43A1*,* MAP2K6*,* ANO10*,* TRPM4*,* RAMP2*,* TRPM6*,* CYB5A*,* GAL*,* ANKH*,***CPT1A***,* SLC26A3*,* PLA2G4A*,* ATP6V0E2*,* ADAMTS8*,* PKP2*,* CLIC5*,* KCNN3*,* SLC7A1*,* CA2*,* CROT*
GOTERM_MF_FAT	Cofactor binding	15	1.22E‐04	***XDH***,* TM7SF2*,* ALDH6A1*,* ECI2*,* KYNU*,* SORD*,* DUOX1*,***CBR3***,* ACADL*,* FMO5*,* SQLE*,***IDH1***,* HHIP*,* GAD1*,* GPT2*
GOTERM_BP_FAT	Monocarboxylic acid metabolic process	22	1.29E‐04	*GGTA1P*,* PAM*,* ECI2*,* KYNU*,* SORD*,* CYP2J2*,* BCKDHB*,***PPARG***,* PDK4*,* CYP26A1*,* ACADL*,***CPT1A***,* ACSM3*,* PLA2G4A*,* ACSM1*,***IDH1***,* UGT8*,* SCD5*,* PCCA*,* DCXR*,* CROT*,* XYLB*
GOTERM_BP_FAT	Organic acid metabolic process	30	1.40E‐04	*PAM*,* KYNU*,* CYP2J2*,* SORD*,***PPARG***,* AGMAT*,* FOLR1*,***IDH1***,* UGT8*,* SCD5*,* GAD1*,* GPT2*,* GGTA1P*,* ECI2*,* ALDH6A1*,* PDK4*,* BCKDHB*,* HGD*,* CYP26A1*,* CYB5A*,* ACADL*,***CPT1A***,* ACSM3*,* ACSM1*,* PLA2G4A*,* QPRT*,* PCCA*,* DCXR*,* CROT*,* XYLB*
GOTERM_BP_FAT	Nitrogen compound transport	26	1.66E‐04	*CALHM1*,* SLC38A4*,* SLC15A2*,* OPRK1*,* TRH*,* SEC14L1*,* KCNS3*,* NOV*,* FOLR1*,***OXCT1***,* TAP2*,* SYBU*,* CHRNA6*,* SLC43A1*,* ABCA12*,* TRPM4*,* ISL1*,* GAL*,* PCLO*,***CPT1A***,* CHGA*,* SLC7A1*,* SYTL4*,* ACE2*,* GNAS*,* CA2*
GOTERM_BP_FAT	Monocarboxylic acid catabolic process	9	1.86E‐04	*ECI2*,* SORD*,* CYP26A1*,* ACADL*,* PCCA*,* CROT*,***CPT1A***,* DCXR*,* XYLB*
GOTERM_BP_FAT	Tissue morphogenesis	23	2.03E‐04	*FRAS1*,* COBL*,* NF2*,* TNC*,* PML*,* NTN4*,* SIX3*,* FZD5*,* ISL1*,* MAGED1*,* ACTG2*,* EYA1*,* EPHA7*,* KRAS*,* PKP2*,* FOLR1*,* WNK4*,* TSC2*,* TFAP2A*,* NFATC4*,* CA2*,* HHIP*,* PRKACB*
GOTERM_BP_FAT	Regulation of secretion	25	2.20E‐04	*PAM*,* OPRK1*,* PML*,* POSTN*,* TLR6*,* TRH*,* KCNS3*,* NOV*,* CASP5*,* WNK4*,***OXCT1***,* SYBU*,* CHRNA6*,* MAP2K6*,* TRPM4*,* GAL*,* ISL1*,* NLRP2*,* PCLO*,***CPT1A***,* CHGA*,* STXBP6*,* SYTL4*,* GNAS*,* CPB2*
GOTERM_BP_FAT	Epithelium development	32	3.93E‐04	*FRAS1*,***XDH***,* COBL*,* TNC*,***PPARG***,* PML*,* CERS3*,* MAGED1*,* KRAS*,* MCOLN3*,* FOLR1*,* WNK4*,* UPK1B*,* NFATC4*,* HHIP*,* PRKACB*,* ABCA12*,* SMAD9*,* NF2*,* NTN4*,* SIX3*,* FZD5*,* GAL*,***CPT1A***,* EYA1*,* EPHA7*,* CLIC5*,* TSC2*,* TFAP2A*,* GNAS*,* CA2*,* LRP4*
GOTERM_BP_FAT	Odontogenesis	9	4.17E‐04	*ASPN*,* PAM*,* NF2*,* SLC24A4*,* TNC*,* COL1A2*,* TFAP2A*,* CA2*,* LRP4*
GOTERM_BP_FAT	Regulation of secretion by cell	23	4.36E‐04	*TRPM4*,* PAM*,* OPRK1*,* PML*,* POSTN*,* TRH*,* TLR6*,* ISL1*,* GAL*,* PCLO*,* NLRP2*,***CPT1A***,* KCNS3*,* CASP5*,* NOV*,* CHGA*,* STXBP6*,***OXCT1***,* SYBU*,* SYTL4*,* GNAS*,* CHRNA6*,* CPB2*
GOTERM_MF_FAT	Coenzyme binding	11	8.04E‐04	*TM7SF2*,***XDH***,* ECI2*,* FMO5*,* ALDH6A1*,* SORD*,* SQLE*,* DUOX1*,***IDH1***,***CBR3***,* ACADL*
GOTERM_BP_FAT	Chemical homeostasis	31	8.72E‐04	*STEAP4*,* SLC39A14*,* ATP1B1*,***PPARG***,* PML*,* CKB*,* PDE6A*,* SLC24A4*,* ANK3*,* TAP2*,***OXCT1***,* WNK4*,* SLC39A8*,* PRKACB*,* ABCA12*,* TRPM4*,* HERPUD1*,* PDK4*,* TRIM24*,* GJB6*,* ACADL*,* ABCG1*,* ACSM3*,* SLC26A3*,* ACSM1*,* PLA2G4A*,* ATP6V0E2*,* PKP2*,* GNAS*,* CA2*,* CPB2*
GOTERM_BP_FAT	Response to endogenous stimulus	42	8.80E‐04	*ASPN*,* PAM*,* KYNU*,* SORD*,* OPRK1*,* TNC*,***PPARG***,* DUOX1*,* PML*,* POSTN*,* GNG11*,* TRH*,* GREM2*,* PEA15*,* KRAS*,* FOLR1*,* GSN*,***OXCT1***,***IDH1***,* CHRNA6*,* PRKACB*,* RAMP2*,* SMAD9*,* STMN2*,* PDK4*,* TRIM24*,* GAL*,* ISL1*,* ABCG1*,* GJB2*,* SLC26A3*,* PLA2G4A*,* ATP6V0E2*,***HMGCS2***,* TSC2*,* COL1A2*,* GNAS*,* CA2*,* BMPR1B*,* HDAC9*,* CROT*,* LRP4*
GOTERM_CC_FAT	Lamellipodium	11	8.99E‐04	*ACTG2*,* SLC39A14*,* NF2*,* SORBS2*,* GSN*,* PLEKHH2*,* STMN2*,* NEDD9*,* IQGAP2*,* ITSN1*,* CTNNA2*
GOTERM_BP_FAT	Secretion by cell	29	.001130969	*PAM*,* NAAA*,* OPRK1*,* PML*,* POSTN*,* TLR6*,* TRH*,* KCNS3*,* CASP5*,* NOV*,***OXCT1***,* SYN2*,* SYBU*,* CREB3L1*,* CHRNA6*,* GAD1*,* ABCA12*,* TRPM4*,* ACTN1*,* ISL1*,* GAL*,* PCLO*,* NLRP2*,***CPT1A***,* CHGA*,* STXBP6*,* SYTL4*,* GNAS*,* CPB2*
GOTERM_BP_FAT	Organ morphogenesis	29	.001210502	*FRAS1*,* ASPN*,* PAM*,* TNC*,* PML*,* MAGED1*,* ACTG2*,* KRAS*,* SLC24A4*,* FOLR1*,* WNK4*,* HHIP*,* NF2*,* SIX3*,* NTN4*,* GJB6*,* FZD5*,* ISL1*,* CTNNA2*,* EYA1*,* PKP2*,* CLIC5*,* COL1A2*,* TFAP2A*,* GNAS*,* CA2*,* BMPR1B*,* CPB2*,* LRP4*
GOTERM_BP_FAT	Regulation of biomineral tissue development	7	.001363066	*ASPN*,* TRPM4*,* PLA2G4A*,* TFAP2A*,* BMPR1B*,* TMEM119*,* ANKH*
GOTERM_BP_FAT	Regulation of protein secretion	16	.001462035	*TRPM4*,* PAM*,* PML*,* POSTN*,* TLR6*,* ISL1*,* TRH*,* NLRP2*,***CPT1A***,* KCNS3*,* NOV*,* CASP5*,***OXCT1***,* SYBU*,* SYTL4*,* GNAS*
GOTERM_CC_FAT	Proteinaceous extracellular matrix	16	.001470486	*FRAS1*,* ASPN*,* HAPLN1*,* TNC*,* OLFML2B*,* NTN4*,* POSTN*,* COL5A3*,* NOV*,* OGN*,* BGN*,* ADAMTS8*,* KAZALD1*,* CCBE1*,* COL1A2*,* TFPI2*
GOTERM_BP_FAT	Nervous system development	52	.00156293	*ATL1*,***PPARG***,* POSTN*,* CKB*,* CASP5*,* CCDC141*,* OGN*,* PRMT1*,* MCOLN3*,* ANK3*,* GSN*,* PRKACB*,* HHIP*,* CDK5RAP2*,* PLCB1*,* ATOH7*,* TNIK*,* STMN2*,* SIX3*,* COL25A1*,* GAL*,* PCLO*,* CTNNA2*,* EYA1*,* CLIC5*,* TFAP2A*,* PAM*,* COBL*,* FRYL*,* TNC*,* KRAS*,* FOLR1*,***OXCT**1*,* NFATC4*,* NDRG2*,* UGT8*,* DCLK1*,* HAPLN1*,* SMAD9*,* NF2*,* NTN4*,* FZD5*,* ISL1*,* EPHA7*,***HMGCS2***,* TSC2*,* MAP2*,* MPPED2*,* HDAC9*,* BMPR1B*,* PHF8*,* LRP4*
GOTERM_BP_FAT	Inorganic ion transmembrane transport	23	.001683334	*CALHM1*,* C15ORF48*,* TRPM4*,* SLC39A14*,* ATP1B1*,* TRPM6*,* OPRK1*,* CYB5A*,* GAL*,* ANKH*,* KCNIP4*,* KCNS3*, *SLC26A3*,* ATP6V0E2*,* ADAMTS8*,* SLC24A4*,* MCOLN3*,* ANK3*,* CLIC5*,* KCNN3*,* TTYH2*,* SLC39A8*,* ANO10*
GOTERM_BP_FAT	Peptide hormone secretion	12	.001842318	*KCNS3*,* TRPM4*,* NOV*,***OXCT1***,* SYBU*,* SYTL4*,* GNAS*,* ISL1*,* TRH*,* GAL*,* PCLO*,***CPT1A***
GOTERM_BP_FAT	Regulation of insulin secretion	10	.001898558	*KCNS3*,* TRPM4*,* NOV*,***OXCT1***,* SYBU*,* SYTL4*,* GNAS*,* ISL1*,* TRH*,***CPT1A***
GOTERM_BP_FAT	Regulation of peptide transport	11	.002159806	*KCNS3*,* TRPM4*,* NOV*,***OXCT1***,* SYBU*,* SYTL4*,* GNAS*,* CA2*,* ISL1*,* TRH*,***CPT1A***
GOTERM_BP_FAT	Xylulose 5‐phosphate metabolic process	3	.002231113	*SORD*,* DCXR*,* XYLB*
GOTERM_BP_FAT	Glucuronate catabolic process to xylulose 5‐phosphate	3	.002231113	*SORD*,* DCXR*,* XYLB*
GOTERM_BP_FAT	Xylulose 5‐phosphate biosynthetic process	3	.002231113	*SORD*,* DCXR*,* XYLB*
GOTERM_BP_FAT	Glucuronate catabolic process	3	.002231113	*SORD*,* DCXR*,* XYLB*
GOTERM_BP_FAT	Cation transport	28	.002320182	*CALHM1*,* C15ORF48*,* SLC38A4*,* SLC39A14*,* STEAP4*,* ATP1B1*,* MFSD3*,* SLC15A2*,* OPRK1*,* PML*,* SEC14L1*, *KCNIP4*,* KCNS3*,* MCOLN3*,* SLC24A4*,* ANK3*, *WNK4*,* SLC39A8*,* CHRNA6*,* ANO10*,* TRPM4*, *RAMP2*,* TRPM6*,* CYB5A*,* GAL*,* ATP6V0E2*,* PKP2*,* KCNN3*
GOTERM_BP_FAT	Peptide secretion	12	.002431887	*KCNS3*,* TRPM4*,* NOV*,***OXCT1***,* SYBU*,* SYTL4*,* GNAS*,* ISL1*,* TRH*,* GAL*,* PCLO*,***CPT1A***
GOTERM_BP_FAT	Morphogenesis of an epithelium	18	.002441073	*FRAS1*,* COBL*,* TNC*,* PML*,* NTN4*,* FZD5*,* MAGED1*,* EPHA7*,* EYA1*,* KRAS*,* FOLR1*,* WNK4*,* TSC2*,* TFAP2A*,* NFATC4*,* CA2*,* PRKACB*,* HHIP*
GOTERM_BP_FAT	Anion transport	17	.002448168	*SLC38A4*,***PPARG***,* TRH*,* ANKH*,***CPT1A***,* SLC26A3*,* PLA2G4A*,* ADAMTS8*,* CLIC5*,* TTYH2*,* SLC7A1*,* WNK4*,* CA2*,* SLC43A1*,* MAP2K6*,* CROT*,* ANO10*
GOTERM_BP_FAT	Epithelial tube morphogenesis	13	.002731156	*COBL*,* TNC*,* PML*,* MAGED1*,* EYA1*,* EPHA7*,* KRAS*,* FOLR1*,* WNK4*,* TSC2*,* NFATC4*,* PRKACB*,* HHIP*
GOTERM_BP_FAT	Signal release	16	.002766541	*TRPM4*,* NAAA*,* OPRK1*,* ISL1*,* GAL*,* TRH*,* PCLO*,***CPT1A***, *KCNS3*,* NOV*,***OXCT1***,* SYN2*,* SYBU*,* SYTL4*,* GNAS*,* GAD1*
GOTERM_BP_FAT	Regulation of hormone secretion	12	.002882484	*KCNS3*,* TRPM4*,* NOV*,* OPRK1*,***OXCT1***,* SYBU*,* SYTL4*,* GNAS*,* ISL1*,* TRH*,* GAL*,***CPT1A***
GOTERM_BP_FAT	Protein localization	55	.002908085	*COPA*,* ATP1B1*,* SLC15A2*,***PPARG***,* VPS37B*,* POSTN*,* SELENBP1*,* TLR6*,* KCNIP4*,* AP1S3*,* NOV*,* CASP5*,* ANK3*,* GSN*,* WNK4*,* TTC21A*,* TRPM4*,* RAMP2*,* TNIK*,* SIX3*,* GAL*,* NLRP2*,* PCLO*,* EYA1*,* HEPACAM*,* STXBP6*,* CLIC5*,* GNAS*,* FRAS1*,* PAM*,* PML*,* RABGAP1L*,* TRH*,* KCNS3*,* TMED3*,***OXCT1***,* TAP2*,* SYBU*,* UGT8*,* DCLK1*,* ABCA12*,* HERPUD1*,* NF2*,* ITGA4*,* FZD5*,* ISL1*,***CPT1A***,* ABCG1*,* ATP6V0E2*,* PKP2*,* KCNN3*,* TSC2*,* SYTL4*,* SNX30*,* LRP4*
GOTERM_BP_FAT	Regulation of transport	44	.002936176	*CALHM1*,* PAM*,* ATP1B1*,* OLFM4*,* OPRK1*,***PPARG***,* PML*,* VPS37B*,* RABGAP1L*,* POSTN*,* TLR6*,* TRH*,* KCNIP4*,* KCNS3*,* CASP5*,* NOV*,* PEA15*,* ANK3*,* WNK4*,***OXCT1***,* SYBU*,* CHRNA6*,* MAP2K6*,* SGIP1*,* ABCA12*,* TRPM4*,* FZD5*,* GAL*,* ISL1*,* NLRP2*,* PCLO*,* ABCG1*,***CPT1A***,* PLA2G4A*,* CHGA*,* STXBP6*,* PKP2*,* CLIC5*,* KCNN3*,* TSC2*,* SYTL4*,* GNAS*,* CA2*,* CPB2*
GOTERM_BP_FAT	Hormone secretion	13	.003111804	*TRPM4*,* OPRK1*,* GAL*,* TRH*,* ISL1*,* PCLO*,***CPT1A***,* KCNS3*,* NOV*,***OXCT1***,* SYTL4*,* SYBU*,* GNAS*
GOTERM_BP_FAT	Protein secretion	17	.003213878	*TRPM4*,* PML*,* POSTN*,* TRH*,* TLR6*,* ISL1*,* GAL*,* NLRP2*,* PCLO*,***CPT1A***,* KCNS3*,* NOV*,* CASP5*,***OXCT1***,* SYBU*,* SYTL4*,* GNAS*
GOTERM_BP_FAT	Morphogenesis of a branching structure	10	.003228966	*MAGED1*,* EYA1*,* EPHA7*,* KRAS*,* TNC*,* PML*,* NTN4*,* NFATC4*,* HHIP*,* FZD5*
GOTERM_BP_FAT	Insulin secretion	9	.003253189	*TRPM4*,* NOV*,***OXCT1***,* SYBU*,* SYTL4*,* ISL1*,* TRH*,* GAL*,* PCLO*
GOTERM_BP_FAT	Monovalent inorganic cation transport	17	.003456998	*C15ORF48*,* TRPM4*,* SLC38A4*,* ATP1B1*,* MFSD3*, *SLC15A2*,* OPRK1*,* CYB5A*,* GAL*,* KCNIP4*,* KCNS3*,* ATP6V0E2*,* SLC24A4*,* PKP2*,* ANK3*,* KCNN3*, *WNK4*
GOTERM_BP_FAT	Regulation of hormone levels	17	.003534129	*TRPM4*,* OPRK1*,* DUOX1*,* CYP26A1*,* TRH*,* ISL1*,* GAL*,* PCLO*,***CPT1A***,* IYD*,* KCNS3*,* NOV*,***OXCT1***,* SYBU*,* SYTL4*,* ACE2*,* GNAS*
GOTERM_CC_FAT	Golgi apparatus	40	.003725799	*COPA*,* PAM*,* STEAP4*,* SLC39A14*,* ATL1*,***PPARG***,* NEDD9*,* RABGAP1L*,* POSTN*,* ST8SIA3*,* TLR6*,* SEC14L1*,* KCNS3*,* AP1S3*,* RNF125*,* OGN*,* GALNT10*,* TMED3*,* ANK3*,* FOLR1*,* SYBU*,* FUT3*,* CALN1*,* NDRG2*,* CDK5RAP2*,* MUC15*,* TRPM4*,* GGTA1P*,* STMN2*,* MUC7*,* FZD5*,* GAL*,* NLRP2*,* ABCG1*,* PLA2G4A*,* BGN*,* CLIC5*,* TSC2*,* TFAP2A*,* GNAS*
GOTERM_MF_FAT	Sulfur compound binding	11	.003932387	*NOV*,* ECI2*,* OGN*,* ALDH6A1*,* ADAMTS8*,* SERPINA5*,* COL25A1*,* POSTN*,* COL5A3*,* ACADL*,* GREM2*
GOTERM_BP_FAT	Hormone transport	13	.004094245	*TRPM4*,* OPRK1*,* GAL*,* TRH*,* ISL1*,* PCLO*,***CPT1A***,* KCNS3*,* NOV*,***OXCT1***,* SYTL4*,* SYBU*,* GNAS*
GOTERM_BP_FAT	Dorsal/ventral pattern formation	7	.00420815	*SIX3*,* HHIP*,* PRKACB*,* BMPR1B*,* FZD5*,* GREM2*,* LRP4*
GOTERM_CC_FAT	Perinuclear region of cytoplasm	22	.004356611	*DYNC1I1*,* CAPN6*,* COBL*,* PAM*,* OLFM4*,* TNIK*,* NF2*,* STMN2*,***PPARG***,* TPD52L1*,* FZD5*,* TPD52*,* CHGA*,* PLA2G4A*,* GSN*,* SORBS2*,* TSC2*,* GNAS*,* CALN1*,* NDRG2*,* CDK5RAP2*,* PRKACB*
GOTERM_BP_FAT	Fatty acid metabolic process	14	.0044624	*GGTA1P*,* ECI2*,* PAM*,* CYP2J2*,***PPARG***,* PDK4*,* ACADL*, ***CPT1A***,* ACSM3*,* PLA2G4A*,* ACSM1*,* SCD5*,* PCCA*,* CROT*
GOTERM_MF_FAT	Active transmembrane transporter activity	14	.004482151	*SLC26A3*,* SLC38A4*,* ATP1B1*,* ATP6V0E2*,* SLC24A4*,* MFSD3*,* SLC15A2*,* TAP2*,* SLC7A1*,* ABCC13*,* ANKH*,* ABCG1*,* ABCA12*,* SLC46A2*
GOTERM_MF_FAT	Ion binding	88	.004564204	*ASPN*,* STEAP4*,* ATP1B1*,* KYNU*,* CYP2J2*,* ATL1*,* ZFP42*,* ZNF530*,***PPARG***,* DUOX1*,* POSTN*,* TPD52*,* ITSN1*,* KCNIP4*,* SLC24A4*,* GSN*,* SERPINA5*,* CCBE1*,* CALN1*,* CHRNA6*,* HHIP*,* PRKACB*,* SCD5*,* PLCB1*,* GPT2*,* NUDT16*,* TRPM6*,* PLD6*,* CYP26A1*,* ACTN1*,* CYB5A*,* PCLO*,* NME7*,* NEBL*,* ZNF233*,* EYA1*,* ADAMTS8*,* CA8*,* CAPN12*,* COL1A2*,* GNAS*,* ADAM18*,* CA2*,* ADAM12*,* PCCA*,* ZNF436*,* FRAS1*,* CAPS*,***XDH***,* PAM*,* SORD*,* YPEL4*,* PML*,* ST8SIA3*,* AGMAT*,* PDE6A*,* RNF125*,* KRAS*,* GALNT10*,* TCEA3*,* FOLR1*,* SORBS2*,* PDE1A*,***IDH1***,* GAD1*,* SMAD9*,* IKZF2*,* PDZRN4*,* VWCE*,* HGD*,* TRIM24*,* ITGA4*,* CSRP2*,* ISL1*,* XPNPEP2*,* ACSM3*,* PAPOLA*,* ACSM1*,* PLA2G4A*,* ZIC4*,* SYTL4*,* ACE2*,* MPPED2*,* HDAC9*,* BMPR1B*,* PHF8*,* CPB2*,* LRP4*
GOTERM_BP_FAT	Regulation of bone mineralization	6	.004761268	*TRPM4*,* PLA2G4A*,* TFAP2A*,* BMPR1B*,* TMEM119*,* ANKH*
GOTERM_BP_FAT	Extracellular matrix organization	13	.005198597	*RAMP2*,* HAPLN1*,* BGN*,* GSN*,* KAZALD1*,* TNC*,* OLFML2B*,* COL1A2*,* CREB3L1*,* POSTN*,* ITGA4*,* COL5A3*,* CPB2*
GOTERM_BP_FAT	Cellular response to chemical stimulus	58	.00526536	*ASPN*,* CYP2J2*,***PPARG***,* DUOX1*,* POSTN*,* NOV*,* ANK3*,* GSN*,* CCBE1*,* CREB3L1*,* PRKACB*,* HHIP*,* PLCB1*,* MAP2K6*,* IFNGR1*,* TRPM4*,* RAMP2*,* STMN2*,* CYP26A1*,* SERPINB9*,* SLC26A3*,* CHGA*,* BGN*,* COL1A2*,* TFAP2A*,* GNAS*,* CA2*,***XDH***,* OPRK1*,* TNC*,* PML*,* GNG11*,* TRH*,* GREM2*,* KRAS*,* FOLR1*,***OXCT**1*,* NFATC4*,* THPO*,* HERPUD1*,* SMAD9*,* PDK4*,* ITGA4*,* TRIM24*,* GJB6*,* ISL1*,***CPT1A***,* GJB2*,* PLA2G4A*,* ACSM1*,* ATP6V0E2*,* GPR37*,***HMGCS2***,* TSC2*,* HDAC9*,* BMPR1B*,* CPB2*,* LRP4*
GOTERM_BP_FAT	Extracellular structure organization	13	.00532233	*RAMP2*,* HAPLN1*,* BGN*,* GSN*,* KAZALD1*,* TNC*,* OLFML2B*,* COL1A2*,* CREB3L1*,* POSTN*,* ITGA4*,* COL5A3*,* CPB2*
GOTERM_MF_FAT	Glycosaminoglycan binding	10	.005386366	*NOV*,* OGN*,* HAPLN1*,* BGN*,* ADAMTS8*,* SERPINA5*,* COL25A1*,* POSTN*,* COL5A3*,* GREM2*
GOTERM_BP_FAT	Positive regulation of stress‐activated MAPK cascade	8	.005588879	***XDH***,* PRMT1*,* TNIK*,* OPRK1*,* TPD52L1*,* TLR6*,* FZD5*,* PLCB1*
GOTERM_BP_FAT	Regulation of peptide hormone secretion	10	.005698819	*KCNS3*,* TRPM4*,* NOV*,***OXCT1***,* SYBU*,* SYTL4*,* GNAS*,* ISL1*,* TRH*,***CPT1A***
GOTERM_BP_FAT	Morphogenesis of a branching epithelium	9	.005744299	*MAGED1*,* EYA1*,* KRAS*,* TNC*,* PML*,* NTN4*,* NFATC4*,* HHIP*,* FZD5*
GOTERM_BP_FAT	Positive regulation of stress‐activated protein kinase signaling cascade	8	.005806735	***XDH***,* PRMT1*,* TNIK*,* OPRK1*,* TPD52L1*,* TLR6*,* FZD5*,* PLCB1*
GOTERM_MF_FAT	NADP binding	5	.006314099	*TM7SF2*,* FMO5*,* DUOX1*,***IDH1***,***CBR3***
GOTERM_BP_FAT	Ion transmembrane transport	26	.006573948	*CALHM1*,* C15ORF48*,* SLC39A14*,* ATP1B1*,* OPRK1*,* KCNIP4*,* KCNS3*,* MCOLN3*,* SLC24A4*,* ANK3*,* TTYH2*,* WNK4*,* SLC39A8*,* CHRNA6*,* ANO10*,* TRPM4*,* TRPM6*,* CYB5A*,* GAL*,* ANKH*,***CPT1A***,* SLC26A3*,* ATP6V0E2*,* ADAMTS8*,* KCNN3*,* CLIC5*
GOTERM_BP_FAT	Regulation of peptide secretion	10	.006609351	*KCNS3*,* TRPM4*,* NOV*,***OXCT1***,* SYBU*,* SYTL4*,* GNAS*,* ISL1*,* TRH*,***CPT1A***
GOTERM_BP_FAT	Response to hormone	25	.00677892	*PAM*,* SORD*,* TNC*,* OPRK1*,***PPARG***,* GNG11*,* TRH*,* KRAS*,***OXCT1***,***IDH1***,* PRKACB*,* RAMP2*,* PDK4*,* TRIM24*,* ISL1*,* GAL*,* ABCG1*,* GJB2*,* PLA2G4A*,* ATP6V0E2*,***HMGCS2***,* TSC2*,* GNAS*,* CA2*,* HDAC9*
GOTERM_BP_FAT	Response to vitamin	7	.006968045	*PLA2G4A*,* KYNU*,* FOLR1*,* GSN*,* TNC*,***PPARG***,* POSTN*
GOTERM_BP_FAT	Homeostatic process	39	.006995908	*STEAP4*,* SLC39A14*,* ATP1B1*,* OPRK1*,***PPARG***,* PML*,* CKB*,* NOV*,* PDE6A*,* PRMT1*,* KRAS*,* SLC24A4*,* ANK3*,* TAP2*,* WNK4*,***OXCT1***,* SYBU*,* SLC39A8*,* PRKACB*,* SGIP1*,* ABCA12*,* TRPM4*,* HERPUD1*,* HMBOX1*,* PDK4*,* TRIM24*,* GJB6*,* ACADL*,* ABCG1*,* ACSM3*,* SLC26A3*,* PLA2G4A*,* ACSM1*,* ATP6V0E2*,* PKP2*,* GNAS*,* CA2*,* CPB2*,* SLC46A2*
GOTERM_BP_FAT	Tube morphogenesis	13	.00712882	*COBL*,* TNC*,* PML*,* MAGED1*,* EYA1*,* EPHA7*,* KRAS*,* FOLR1*,* WNK4*,* TSC2*,* NFATC4*,* PRKACB*,* HHIP*
GOTERM_BP_FAT	Response to organic substance	61	.007992486	*ASPN*,* KYNU*,***PPARG***,* DUOX1*,* POSTN*,* TLR6*,* GSN*,* CCBE1*,* CREB3L1*,* CHRNA6*,* PRKACB*,* HHIP*,* PLCB1*,* MAP2K6*,* IFNGR1*,* TRPM4*,* RAMP2*,* STMN2*,* GAL*,* SLC26A3*,* BGN*,* SQLE*,* COL1A2*,* TFAP2A*,* GNAS*,* CA2*,* CROT*,***XDH***,* PAM*,* SORD*,* OPRK1*,* TNC*,* PML*,* GNG11*,* TRH*,* GREM2*,* PEA15*,* KRAS*,* FOLR1*,***OXCT1***,***IDH1***,* THPO*,* HERPUD1*,* SMAD9*,* PDK4*,* ITGA4*,* TRIM24*,* GJB6*,* FZD5*,* ISL1*,***CPT1A***,* ABCG1*,* GJB2*,* PLA2G4A*,* ATP6V0E2*,***HMGCS2***,* TSC2*,* HDAC9*,* BMPR1B*,* CPB2*,* LRP4*
GOTERM_CC_FAT	Cell leading edge	14	.008364418	*COBL*,* SLC39A14*,* NF2*,* STMN2*,* NEDD9*,* IQGAP2*,* ACTN1*,* ITSN1*,* CTNNA2*,* ACTG2*,* GSN*,* SORBS2*,* PLEKHH2*,* GNAS*
GOTERM_BP_FAT	Regulation of peptidase activity	14	.008369752	***XDH***,* SERPINB9*,* HERPUD1*,* EPHA7*,* CARD16*,* GSN*,* SERPINA5*,***PPARG***,* PML*,* CST1*,* NLRP2*,* TFPI2*,* WFDC2*,* PI15*
GOTERM_BP_FAT	Macromolecule localization	59	.009247219	*COPA*,* ATP1B1*,* SLC15A2*,***PPARG***,* VPS37B*,* POSTN*,* SELENBP1*,* TLR6*,* KCNIP4*,* CASP5*,* NOV*,* AP1S3*,* ANK3*,* GSN*,* SERPINA5*,* WNK4*,* TTC21A*,* MAP2K6*,* TRPM4*,* RAMP2*,* TNIK*,* SIX3*,* GAL*,* NLRP2*,* PCLO*,* EYA1*,* HEPACAM*,* STXBP6*,* CLIC5*,* GNAS*,* CROT*,* FRAS1*,* PAM*,* PML*,* RABGAP1L*,* TRH*,* KCNS3*,* TMED3*,* TAP2*,***OXCT1***,* SYBU*,* UGT8*,* DCLK1*,* ABCA12*,* HERPUD1*,* NF2*,* ITGA4*,* FZD5*,* ISL1*,***CPT1A***,* ABCG1*,* PLA2G4A*,* ATP6V0E2*,* PKP2*,* KCNN3*,* TSC2*,* SYTL4*,* SNX30*,* LRP4*
GOTERM_BP_FAT	Glucuronate metabolic process	4	.009462291	*SORD*,* UGT8*,* DCXR*,* XYLB*
GOTERM_BP_FAT	Regulation of fatty acid oxidation	4	.009462291	*PDK4*,***PPARG***,* ACADL*,***CPT1A***
GOTERM_BP_FAT	Uronic acid metabolic process	4	.009462291	*SORD*,* UGT8*,* DCXR*,* XYLB*
GOTERM_BP_FAT	Response to estrogen	6	.009528125	*SERPINB9*,* OPRK1*,***PPARG***,* TRIM24*,* CA2*,* GAL*
GOTERM_BP_FAT	Axon development	15	.009632965	*COBL*,* ATL1*,* TNC*,* NTN4*,* COL25A1*,* ISL1*,* CTNNA2*,* OGN*,* EPHA7*,* KRAS*,* FOLR1*,* ANK3*,* BMPR1B*,* LRP4*,* DCLK1*
GOTERM_BP_FAT	Organonitrogen compound catabolic process	13	.009943818	***XDH***,* NUDT16*,* ALDH6A1*,* KYNU*,* BCKDHB*,* HGD*,* ACADL*,* OGN*,* BGN*,* PDE1A*,* ACE2*,* GAD1*,* GPT2*

MAPK, mitogen‐activated protein kinase; NADP, nicotinamide adenine dinucleotide phosphate. Bold denotes gene names which are picked up in the results and discussion.

In addition, several of the down‐regulated genes (peroxisome proliferator‐activated receptor γ [*PPAR‐*γ], xanthine dehydrogenase [*XDH*], carbonyl reductase 3 [*CBR3*], isocitrate dehydrogenase 1 [*IDH1*], and *CPT1*) fell under the GO term “oxidation‐reduction process” (Tables 4 and S1). These genes have essential roles in the response to oxidative stress.^15^, ^16^, ^17^, ^18^, ^19^, ^20^, ^21^, ^22^, ^23^, ^24^, ^25^

Two KEGG pathways that have a strong association with the down‐regulated genes are “butanoate metabolism” and “metabolic pathways” (Tables 5 and S2). These pathways include *HMGCS2, OXCT1*,* XDH*,* IDH1*, and *CBR3*.

**Table 5:** Kyoto Encyclopedia of Genes and Genomes pathway analysis for the genes that were down‐regulated in the thin endometrium

Term	Count	*P* value	Gene
Butanoate metabolism	5	.001114534	*ACSM3*,* ACSM1*,***HMGCS2***,***OXCT1***,* GAD1*
Metabolic pathways	34	.005119787	*TM7SF2*,***XDH***,* KYNU*,* SORD*,* CYP2J2*,* CERS4*,* CERS3*,* AGMAT*,* CKB*,* GALNT10*,* FUT3*,***IDH1***,* UGT8*,* PLCB1*,* GAD1*,* GPT2*,* ALDH6A1*,* BCKDHB*,* HGD*,* ALDH3B2*,* CYP26A1*,***CBR3***,* ACADL*,* NME7*,* ACSM3*,* PLA2G4A*,* ACSM1*,* ATP6V0E2*,***HMGCS2***,* SQLE*,* QPRT*,* PCCA*,* DCXR*,* XYLB*
Thyroid hormone synthesis	6	.007157684	*ATP1B1*,* CREB3L1*,* GNAS*,* PRKACB*,* PLCB1*,* IYD*
Valine, leucine, and isoleucine degradation	5	.00867887	*ALDH6A1*,***HMGCS2***,***OXCT1***,* BCKDHB*,* PCCA*
Amoebiasis	7	.01007625	*SERPINB9*,* COL1A2*,* ACTN1*,* GNAS*,* PRKACB*,* COL5A3*,* PLCB1*
Serotonergic synapse	7	.012474846	*PLA2G4A*,* CYP2J2*,* KRAS*,* GNG11*,* GNAS*,* PRKACB*,* PLCB1*
Salivary secretion	6	.016558258	*ATP1B1*,* CST1*,* GNAS*,* MUC7*,* PRKACB*,* PLCB1*
Protein digestion and absorption	6	.018126807	*ATP1B1*,* COL1A2*,* ACE2*,* COL5A3*,* CPB2*,* XPNPEP2*
GnRH signaling pathway	6	.020659132	*PLA2G4A*,* KRAS*,* GNAS*,* PRKACB*,* PLCB1*,* MAP2K6*
Pancreatic secretion	6	.022469965	*SLC26A3*,* ATP1B1*,* GNAS*,* CA2*,* PLCB1*,* CPB2*
Inflammatory mediator regulation of TRP channels	6	.027439494	*PLA2G4A*,* CYP2J2*,* GNAS*,* PRKACB*,* PLCB1*,* MAP2K6*
Glucagon signaling pathway	6	.028510884	*PRMT1*,* CREB3L1*,* GNAS*,* PRKACB*,* PLCB1*,* CPT1A*
Melanogenesis	6	.029608533	*KRAS*,* CREB3L1*,* GNAS*,* PRKACB*,* FZD5*,* PLCB1*
Arrhythmogenic right ventricular cardiomyopathy	5	.034477703	*PKP2*,* SGCD*,* ACTN1*,* ITGA4*,* CTNNA2*
Gastric acid secretion	5	.037626762	*ATP1B1*,* GNAS*,* PRKACB*,* CA2*,* PLCB1*
Vasopressin‐regulated water reabsorption	4	.040391867	*DYNC1I1*,* CREB3L1*,* GNAS*,* PRKACB*
Endocrine and other factor‐regulated calcium reabsorption	4	.04274525	*ATP1B1*,* GNAS*,* PRKACB*,* PLCB1*
Cholinergic synapse	6	.043457379	*KRAS*,* CREB3L1*,* GNG11*,* PRKACB*,* CHRNA6*,* PLCB1*

GnRH, gonadotropin‐releasing hormone; TRP, transient receptor potential. Bold denotes gene names which are picked up in the results and discussion.

## 4 Discussion

### 4.1 Up‐regulated genes in the thin endometrium

Although a thin endometrium is known to be involved in implantation failure, the mechanism has not been elucidated. The authors recently found that a high level of blood flow impedance of the uterine radial artery underlies a thin endometrium.^4^ The present study investigated the cause of implantation failure in the thin endometrium by using genome‐wide mRNA expression analysis. Hierarchical clustering and a PCA demonstrated that the thin endometrium and the control endometrium clearly had different mRNA expression profiles, suggesting that aberrant gene expression is involved in implantation failure in a thin endometrium.

The GO analyses showed that the up‐regulated genes in the thin endometrium included a number of genes that are related to immunity. In fact, a KEGG pathway analysis indicated that a number of genes related to natural killer cell cytotoxicity are up‐regulated in thin endometria, suggesting the presence of a cytotoxic condition. Aberrant immunological factors play roles in recurrent miscarriage and implantation failure.^6^, ^26^, ^27^ Interestingly, 56.6% of the patients who experienced embryo implantation failures showed local immune overactivation in the endometrium at the mid‐luteal phase.^6^ An influx of immune cells and a switch of local immunity from the adaptive (Th1) type to the innate (Th2) type have been observed during the implantation window.^6^, ^28^ The Th2 cytokines allow the development of local mechanisms that promote immunotrophism and also down‐regulate the inflammation and cytotoxic pathways.^6^, ^29^ This immune switch, from a Th1 pro‐inflammatory environment to a Th2 anti‐inflammatory environment, is fundamental to the establishment of local maternal tolerance and is crucial for implantation. In this period, uterine natural killer (uNK) cells, together with macrophages and dendritic cells, increase in the endometrium and have a significant role in innate (Th2) immunity.^30^ Unlike peripheral natural killer cells, the uNK cells are not cytotoxic and their main biological functions are to produce immunotrophic and angiogenic cytokines. The activation of adequate uNK cells is important for maternal tolerance during the implantation window.^31^, ^32^, ^33^ However, once the uNK cells are highly activated, a Th1 pro‐inflammatory condition is induced, with the local production of *IFN‐*γ and *TNF‐*α.^34^, ^35^ The *IFN‐*γ and *TNF‐*α activate the uNK cells to become cytotoxic.^34^, ^35^ In fact, the present study showed that the *IFN‐*γ, *FASLG*,* GZMB*,* and TNF‐*α‐induced genes, such as *TNFAIP2* and *TNFAIP6*, were up‐regulated and that natural killer cell cytotoxicity was elevated in the thin endometrium. These results suggest that aberrant overactivation of the uNK cells and a cytotoxic/Th1 pro‐inflammatory environment are present in a thin endometrium, which is associated with implantation failure. However, it is still unknown how impaired blood flow is associated with the aberrant immunity.

### 4.2 Down‐regulated genes in the thin endometrium

The GO analyses indicated that the down‐regulated genes included a number of genes related to catabolic processes, which are essential in breaking down large molecules, such as polysaccharides, lipids, and proteins, into smaller units, such as monosaccharides, fatty acids, and amino acids. These small units are used to synthesize acetyl‐CoA, which is needed to produce adenosine 5′‐triphosphate in the citrate cycle. Acetyl‐CoA is also used for the synthesis of ketone bodies, which can be an energy source. The KEGG pathway analysis showed that the genes related to butanoate metabolism were down‐regulated in the thin endometrium. Genes, such as *CPT1*,* HMGCS2*, and *OXCT1*, are essential for generating acetyl‐CoA and ketone bodies in butanoate metabolism.^13^, ^14^, ^15^ Butanoate is a substrate that is used to generate energy in both aerobic and anaerobic processes. The present findings suggest that energy synthesis in the cell is impaired in the thin endometrium. The deficiency of energy could be associated with cellular dysfunction in the endometrium, resulting in implantation failure.

The GO analyses also identified a number of genes related to oxidation‐reduction processes. These genes included *PPAR‐*γ, *XDH*,* CBR3*,* IDH1*, and *CPT1*, which have essential roles in the cellular responses to oxidative stress. The activation of *PPAR‐*γ is an important factor in the protection against oxidative stress in cells, such as vascular endothelial cells and cardiomyocytes.^16^, ^17^, ^20^, ^21^, ^24^, ^25^ Xanthine dehydrogenase reduces age‐related oxidative stress in tissues and immune cells.^23^ Carbonyl reductase 3 is regulated via *NRF2*‐dependent signaling pathways and helps to alleviate oxidative stress.^18^ Isocitrate dehydrogenase 1 acts as an antioxidant in melanocytes,^19^ and when mutated, it sensitizes cells to oxidative stress.^36^ Carnitine palmitoyltransferase I is involved in mitochondrial beta‐oxidation of long‐chain fatty acids.^15^ The inhibition of *CPT1* leads to the generation of reactive oxygen species.^22^ Oxidative stress in the endometrium has been associated with failures of embryo implantation and embryo development.^37^ The fact that these anti‐oxidative stress genes were down‐regulated in the thin endometrium suggests that a decreased response to oxidative stress is associated with implantation failure.

In conclusion, the present study revealed that the thin endometrium possesses an aberrant Th1‐pro‐inflammatory/Th2‐anti‐inflammatory balance and increased cytotoxic condition and that a protective response to oxidative stress is impaired. These aberrant molecular mechanisms in the thin endometrium might be associated with implantation failure. These findings could lead to better treatments for patients with implantation failure as a result of a thin endometrium.

## Disclosures

*Conflict of interest*: The authors declare no conflict of interest. *Human Rights*: The study protocol was reviewed and approved by the Institutional Review Board of Yamaguchi University Graduate School of Medicine. Informed consent was obtained from the participants before the collection of any sample. All the experiments that involved the handling of human tissues were performed in accordance with the tenets of the Declaration of Helsinki.
